# Association between psychiatric symptoms with multiple peripheral blood sample test: a 10-year retrospective study

**DOI:** 10.3389/fpsyt.2024.1481006

**Published:** 2024-12-09

**Authors:** Jianqing Qiu, Cheng Yu, Yalan Kuang, Yao Hu, Ting Zhu, Ke Qin, Wei Zhang

**Affiliations:** ^1^ West China Biomedical Big Data Center, West China Hospital, Sichuan University, Chengdu, China; ^2^ Medical Big Data Center, Sichuan University, Chengdu, China; ^3^ School of Computer Science and Engineering, University of Electronic Science and Technology of China, Chengdu, China; ^4^ Mental Health Center and Psychiatric Laboratory, the State Key Laboratory of Biotherapy, West China Hospital of Sichuan University, Chengdu, China; ^5^ Huaxi Brain Research Center, West China Hospital of Sichuan University, Chengdu, China

**Keywords:** psychiatric symptoms, research domain criteria (RDoC), peripheral blood sample test, inflammation, natural language processing (NLP)

## Abstract

**Background:**

Psychiatric illness is thought to be a brain somatic crosstalk disorder. However, the existing phenomenology-based Diagnostic and Statistical Manual of Mental Disorders, Fifth Edition (DSM-5) diagnostic framework overlooks various dimensions other than symptoms. In this study, we investigated the associations between peripheral blood test indexes with various symptom levels of major depressive disorder (MDD), bipolar disorder (BD), and schizophrenia (SCZ) to explore the availability of peripheral blood test indexes.

**Methods:**

We extracted cases diagnosed with MDD, BD, and SCZ at West China Hospital from 2009 to 2021, translated their main complaints into Research Domain Criteria (RDoC) symptom severity scores using nature language processing (NLP), and collected their detailed psychiatric symptoms and peripheral blood test results. Then, generalized linear models were performed between seven types of peripheral blood test values with their transformed RDoC scores and detailed symptom information adjusted for age, gender, smoking, and alcohol history.

**Results:**

Several inflammatory-related indexes were strongly associated with the negative valence system (NVS) domain (basophil percentage adjusted *β* = 0.275, lymphocyte percentage adjusted *β* = 0.271, monocyte percentage adjusted *β* = 0.223, neutrophil percentage adjusted *β* = −0.310, neutrophil count adjusted *β* = −0.301, glucose adjusted *β* = −0.287, leukocyte count adjusted *β* = −0.244, NLR adjusted *β* = −0.229, and total protein adjusted *β* = −0.170), the positive valence system (PVS) domain (monocyte percentage adjusted *β* = 0.228, basophil count adjusted *β* = 0.176, and glutamyl transpeptidase adjusted *β* = 0.171), and a wide range of mood, reward, and psychomotor symptoms. In addition, glucose, urea, urate, cystatin C, and albumin showed considerable associations with multiple symptoms. In addition, based on the direction of associations and the similarity of symptoms in terms of RDoC thinking, it is suggested that “positive” mood symptoms like mania and irritability and “negative” mood symptoms like depression and anxiety might be on a continuum considering their opposite relationships with similar blood indexes.

**Limitations:**

The cross-sectional design, limited symptoms record, and high proportion of missing values in some other peripheral blood indexes limited our findings.

**Conclusion:**

The proportion of high inflammatory indexes in SCZ was relatively high, but in terms of mean values, SCZ, BD, and MDD did not differ significantly. Inflammatory response showed a strong correlation with NVS, PVS, and a range of psychiatric symptoms especially mood symptoms, psychomotor symptoms, and cognitive abilities.

## Highlights

- Nature language processing was used to translate patients’ main complaint into five RDoC domain scores to characterize their symptom severity in one certain domain.
- Significant associations between the negative valence domain, the positive valence domain, and a series of peripheral inflammatory factors were found.
- Several significant associations between various detailed mood-related, reward-related, and sensorimotor symptoms and inflammatory factors and other peripheral factors were found.

## Introduction

As Thomas R. Insel pointed out, the “psychiatric illness” syndrome is thought to be a brain disorder interacting between the nervous system and other systems (1). It is suggested that biological changes in the nervous system might happen in mental diseases. For example, the T-tau (tubulin-associated unit) protein, P-tau protein, and amyloid-*β*42 (A*β*42) proved to be significant biomarkers in Alzheimer’s disease (2). However, few biomarkers were proposed in mood-related disorders.

As is already known, the nervous system could regulate heart rate, blood pressure, and breath rhythm through the sympathetic and parasympathetic systems (3). Beyond its autonomic functions, the brain can act as an effect modifier, allowing these systems to be differentially regulated or dysregulated. This variability can be assessed for deviations from normalcy, which might correspond to various psychiatric disorders. These regulatory relationships have been demonstrated in many studies, such as the relationship between depression and heart rate variability (4), with respiratory rhythm (5) and blood pressure (6). On the other hand, the brain could regulate the neuroendocrine response by secreting hormones through neuroendocrine systems such as the hypothalamic–pituitary–adrenal (HPA) axis. This relationship has also been suggested in several studies (7, 8); it has been shown that an overactivity of the HPA axis was detected in patients with major depressive disorder (MDD).

In addition to the aforementioned studies, a growing number of new hypotheses of mental illness have been proposed. These include (1) the brain–gut axis hypothesis: bidirectional biochemical signaling that occurs between the intestinal mucosa and the central nervous system (9, 10) might mediate a strong link between the gut microflora and the brain; (2) the liver–brain axis hypothesis: a range of factors in the liver might mediate responses to the brain (11); and (3) the lung–brain axis hypothesis: the lung microbiota could modulate brain autoimmunity (12, 13).

All the aforementioned studies have demonstrated that psychiatric illness is a complex disease interaction between the brain and the body. On one hand, several studies have indicated that various types of metabolites in other organs could contribute to the development of mental diseases by impacting the brain’s immunity environment. On the other hand, certain studies have highlighted that the brain could influence other organs through pathways like the HPA axis. In some empirical studies, considerable associations between the PRS score of certain psychiatric diseases and peripheral blood indicators could be presented (14), and the role of inflammation was highlighted. Moreover, some studies have shown a stronger association between cognitive function and multiple peripheral blood indicators in populations with MDD, schizophrenia (SCZ), and bipolar disorder (BD) (15–17). For example, a range of cytokines including CCL, CXCL, G-CSF, GM-CSF, IFN-γ, and IL were found to be significantly increased in MDD patients (18). Additionally, BD and SCZ have been suggested to be associated with increased levels of peripheral inflammatory markers such as IL-1*β*, sIL-2r, IL-6, and TNF-*α* as well (19, 20).

However, in recent years, Diagnostic and Statistical Manual of Mental Disorders, Fifth Edition (DSM-5) diagnosis based on symptom phenomenology has been recognized as heterogeneous. That is, different DSM-5 disease diagnoses like MDD and BD proved to be sharing the same genes (21) and similar symptoms (22, 23), whereas the same DSM-5 diagnosis often presents different genes and symptoms, suggesting that the heterogeneity that broadly existed in current psychiatric DSM-5 disorders might be neglected and should be considered in a more fine-grained level. A series of inflammatory associations have been reported in MDD, BD, and SCZ, suggesting that similar immune mechanisms might exist across these diseases. However, even within the same diagnosis, various patterns of association have been observed concerning the same cytokine. For instance, increased levels of C-reactive protein and TNF-*α* have been found to be associated only with atypical symptoms of MDD (24). Additionally, it has been suggested that IL-1*β* and IL-6 were only associated with suicidal MDD (25). Similarly, leukocyte, neutrophil, and monocyte counts, as well as monocyte-to-lymphocyte and neutrophil-to-lymphocyte ratios, were higher in patients with BD experiencing mania compared to those exhibiting depressive symptoms (26). In total, a specific inflammatory pattern in blood samples was found to be associated with particular symptoms, even within the same diagnosis. As Beurel et al. suggested, serotonin was linked to sadness and appetite, dopamine was associated with motivation and sociability, and norepinephrine was connected to energy (18).

More specifically, the symptoms rather than the diagnosis were suggested to be more directly reflected by peripheral blood indicators. For example, Marsland et al. noted a significant association between acute laboratory stress and increased circulating IL-6, IL-1*β*, IL-10, and TNF-*α* (27). In contrast, chronic stress was found to be associated with elevated levels of plasma IL-6 (28). As Goldsmith et al. pointed out, discrete neural circuits were associated with various corresponding cross-diagnostic symptoms such as reduced motivation, decreased motivation, and psychomotor slowing in all MDD, BD, and SCZ populations. Therefore, as these symptoms were associated with distinct neural circuits, various circulating inflammatory cytokines (e.g., IL-1, IL-6, TNF, and IFN) played various kinds of roles in the dysfunctional connectivity within various circuits (29, 30).

As the Research Domain Criteria (RDoC) propose, the characteristics of mental disorders should be considered at a more fine-grained phenomenological level based on a clear neurological basis. As the National Institute of Mental Health (NIMH) suggested, the RDoC treated psychopathological system as six domains, namely, the negative valence system (NVS) domain, the positive valence system (PVS) domain, the cognitive system domain, the social process system domain, the arousal system domain, and the sensorimotor system domain (31). NVS was primarily responsible for responses to aversive situations or context, such as fear, anxiety, and loss. PVS was defined as primarily responsible for responses to positive motivational situations or contexts, such as reward seeking, consummatory behavior, and reward/habit learning. Moreover, the cognitive, social process, arousal, and sensorimotor systems were defined as their names suggest. According to RDoC thinking, symptoms and RDoC scores could better reflect the heterogeneity of mental illness. Considering most studies have explored the associations between DSM diagnosis or several specific symptoms with inflammatory markers, based on the consideration of heterogeneity, our study extracted patients diagnosed with MDD, BD, and SCZ from 2009 to 2021 in West China Hospital, and collected information containing their main complaints, detailed symptoms in terms of RDoC systems, and peripheral blood biomarkers reflecting various physiological systems, and explored the association between peripheral blood biomarkers with different DSM-5 diagnoses, transdiagnostic RDoC scores based on the free text of main complaints, and various symptoms.

## Materials and methods

The samples were taken from the electronic medical record system of West China Hospital of Sichuan University. The electronic medical record system (HIS) was first implemented at the end of 2008 at West China Hospital, through which physicians recorded admissions and discharges. In 2008, all departments in the hospital adopted the electronic medical record system integrated with the HIS and the LIS, and this was considered the starting time for data extraction. The Biomedical Research Ethics Committee of West China Hospital of Sichuan University has approved our study (No. HX-IRB-AF-21-V3.0).

**Figure 1:**
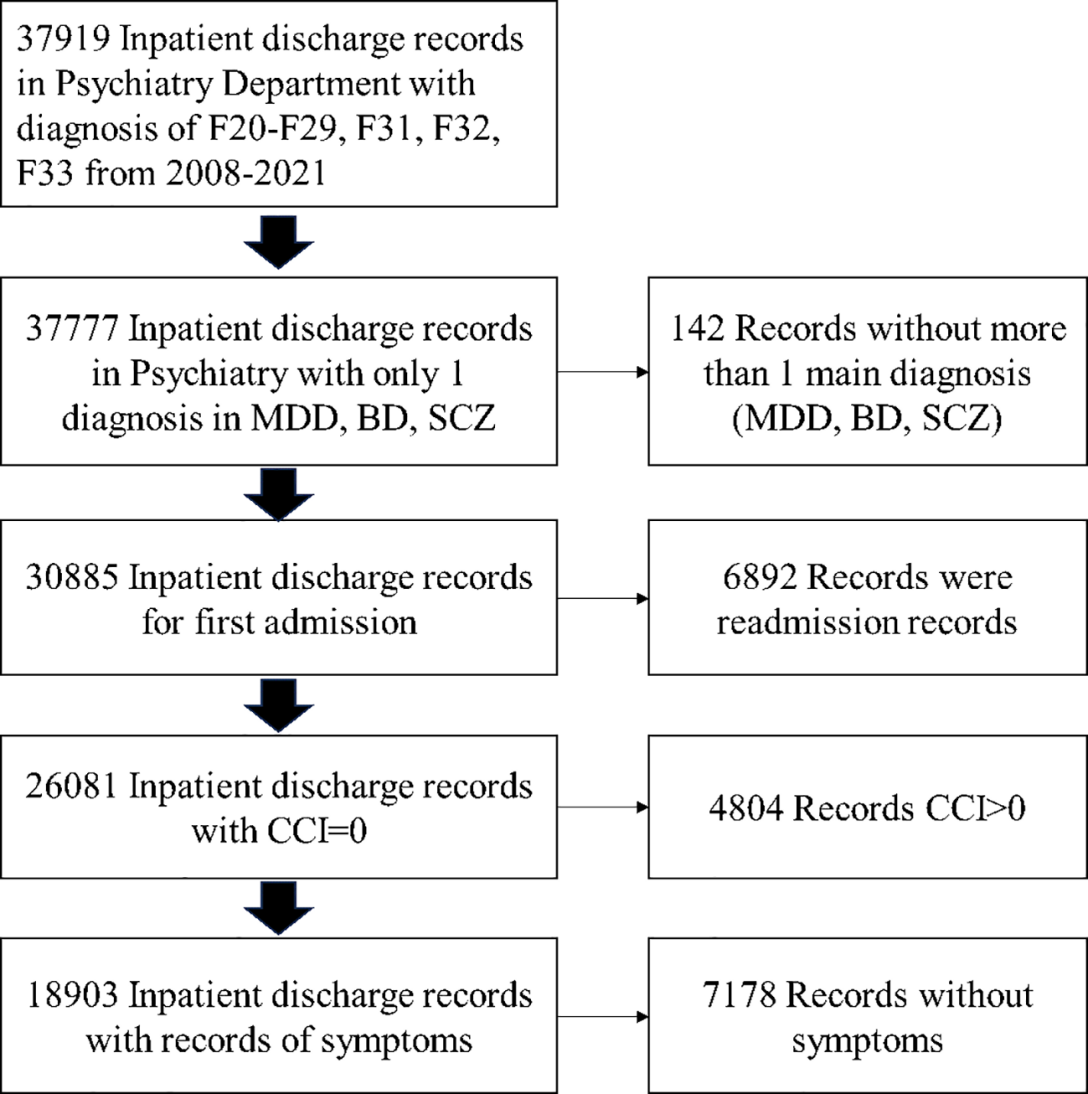
Flow diagram of the subjects’ inclusion/exclusion process.

The initial sample came from a research database created by extracting information from the electronic medical records of West China Hospital, and the inclusion/exclusion flowchart is shown in **Figure 1**. The patients were diagnosed as having MDD (ICD-10: F32, F33), BD (ICD-10: F31), and SCZ (ICD-10: F20-F29) who were hospitalized and discharged at least once between 1 January 2009 and 31 August 2021. To dismiss the effects of substance use and chronic illnesses, the patients with multiple diagnoses of MDD, BD, and SCZ; Carlson Comorbidity Index (CCI) > 0; and first admission were excluded.

**Figure 2:**
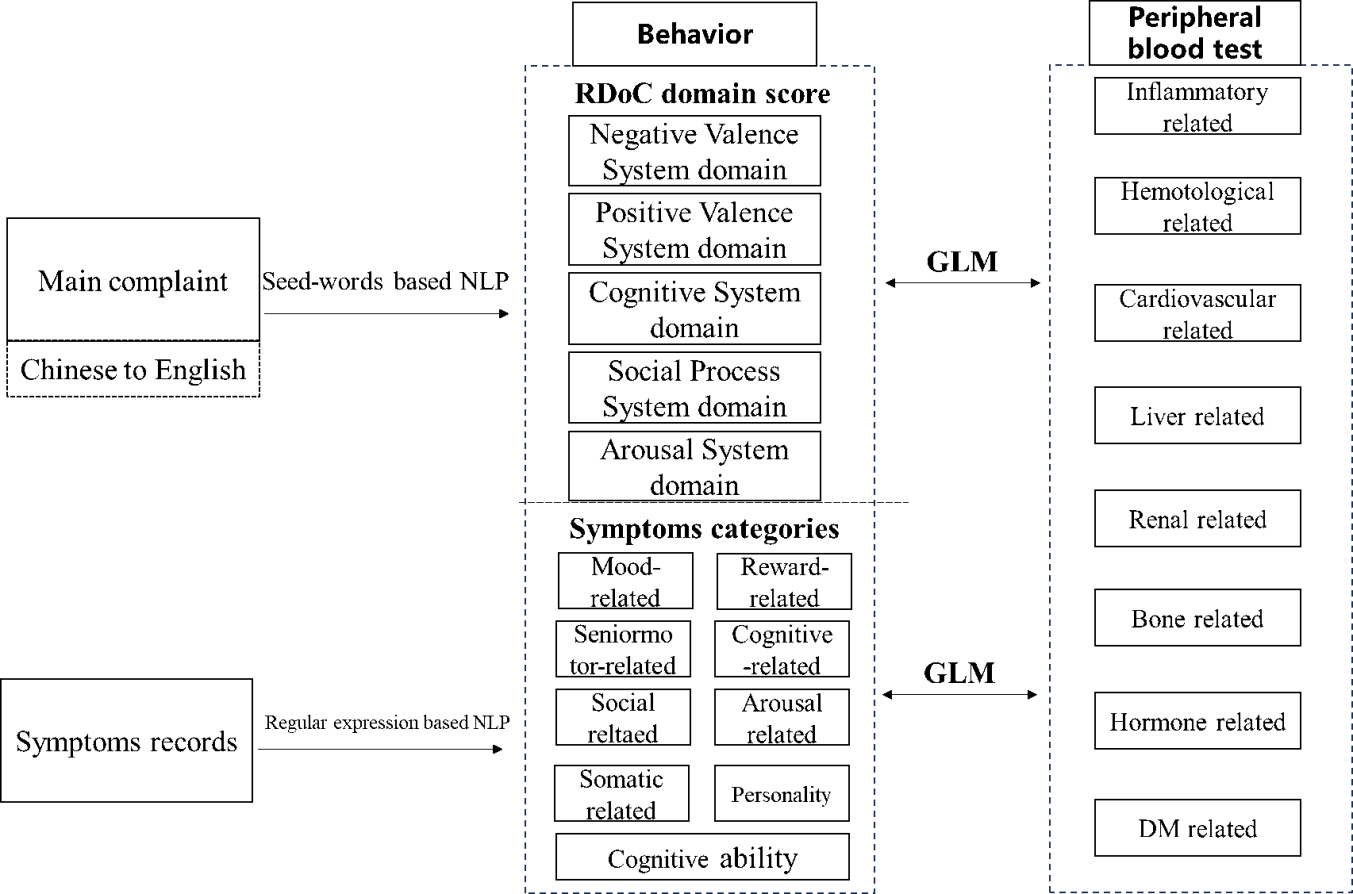
Overall workflow of RDoC domain score calculation, symptom categories generation, and association analysis.

The overall study design is shown in **Figure 2**; we transformed the main complaint to five RDoC domain scores, calculated the patient’s CCI, preprocessed the patient’s symptom information and peripheral blood test, and then performed the association analysis.

### RDoC domain score and CCI calculation

As NIMH suggested (31), the RDoC initially divided the psychological function into five domains: negative valence system (NVS), positive valence system (PVS), cognitive systems, systems for social process, and arousal/modulatory systems.

The algorithm suggested by McCoy et al. (32) was employed to compute the RDoC score using main complaints from the electronic medical records. In summary, the score could be computed by utilizing a collection of words from the corpus and determining the frequency of specific RDoC tokens that are present in the document. The RDoC token list was established by psychiatric professionals who collaborated in the NIMH RDoC Working Group. In the above scenario, if a prepared list consists of 10 tokens and an individual’s document contains 2 of those tokens, the individual would be assigned a score of 2/10.

The NIMH RDoC Working Group has made their specified terminology list freely accessible on the Internet (32) at the following URL: https://github.com/thmccoy/cqh-dimensional-phenotyper. Given that the algorithm was designed for English text, a transformer-based algorithm was utilized to translate original EMR text in Chinese into English. This algorithm could be conducted in an offline environment, and the code is available at https://github.com/xiliuya/trans_rpc.

The Charlson Comorbidity Index (CCI) was employed as an exclusion criteria. As a medical instrument, it is widely utilized to forecast the probability of mortality in individuals with diverse comorbidities, such as cardiovascular disease, acquired immunodeficiency syndrome (AIDS), or cancer. It has a total of 17 kinds of comorbidities (33). Each patient’s ICD diagnosis list was used for calculating the CCI using the *comorbidity* package.

### Symptom records

Symptoms were recorded by clinicians in free text, which we processed into “present/absent” or “good/bad” categories using a regular expression method. According to the RDoC framework, we classified these symptoms into mood-related, reward-related, cognitive-related, social-related, arousal-related, sensorimotor-related, and cognitive abilities. Details are presented in **Supplementary Table S1**.

### Peripheral blood test

Peripheral blood tests were performed by the hospital laboratory department and recorded in the HIS system as numerical values, as well as high, low, or normal classifications, respectively. Those variables were classified according to their attributes as inflammatory related, hematological related, cardiovascular related, liver related, renal related, bone related, and DM related. Details are presented in **Supplementary Table S2**. The complete data set observation method was used for missing values. Considering many blood indicators had missing values, we kept variables with missing values <5%.

### Statistical analysis

For comparing the distribution of peripheral blood test among different DSM-5 diagnoses, ANOVA was applied to compare the mean of their individual blood indexes. Generalized linear models (GLMs) were utilized to explore the possible associations between each RDoC score and a series of blood test indexes by adjusting variables such as gender, age, smoking, and alcohol history. Regarding the detailed symptom information, logit models adjusted for gender, age, smoking, and alcohol history were used to explore the association between blood test indexes and detailed symptoms. To make coefficients comparable, all the blood test indexes were scaled. Python 5.10 was used to translate the Chinese text, calculating five RDoC domains and used R 3.6.3 to perform statistical analysis. All the effect sizes and *p*-values of associations are presented in the **Supplementary Materials** and displayed in heatmaps.

## Results

**Supplementary Table S3** shows the distribution of individual symptoms characterized in MDD, BD, and SCZ. Few of the symptoms investigated were unique to only one disease among MDD, BD, and SCZ. This demonstrates that BD, MDD, and SCZ overlap each other within similar symptoms. In particular, MDD had a higher proportion of *anxiety*, *depression*, *concern health*, *feeling of unworthiness*, *feeling of hopelessness*, *feeling of helplessness*, *decreased interest*, *hypoactivity*, *reduced sleep*, *early awakening*, *sweating, palpitation*, and *somatic symptoms*. In contrast, BD had a higher proportion of *irritability*, *mania*, *provocation*, *increased appetite*, *money wasting*, *weight change*, and *hyperactivity*, whereas SCZ had a lower proportion of symptoms than MDD and BD except for *hallucination* and *delusion*.

**Supplementary Table S4** shows the distribution of RDoC scores for the three DSM-5 disorders. Among them, MDD was higher in the NVS domain, BD was higher in the PVS domain, and SCZ was higher in the cognitive domain and social process domain. This result is also consistent with previous studies (34).

**Supplementary Table S5** and **Table 1** show the distribution of individual blood tests in the MDD, BD, and SCZ populations. It is worth noting that among MDD, BD, and SCZ patients discharged, there was a higher proportion of lower eosinophils (12%), higher proportion of higher neutrophils (12%), and higher proportion of high NLR cells (12%) in SCZ. However, the means of three diagnosis were similar, except for the fact that urate was much lower in MDD (295.9).

**Table 1:** The characteristics of peripheral blood test in different DSM-5 categories.

	Overall (*N* = 26,081)	BD (*N* = 3,258)	MDD (*N* = 13,075)	SCZ (*N* = 9,748)	*p*-value
**Basophil percentage (%)**	0.48 (0.30)	0.50 (0.30)	0.51 (0.31)	0.43 (0.30)	**<0.001**
**CRP (mg/L)**	14.80 (33.40)	13.40 (24.70)	12.10 (28.40)	22.30 (45.20)	**<0.001**
**Eosinophil percentage (%)**	2.31 (2.11)	2.50 (2.07)	2.41 (2.13)	2.11 (2.09)	**<0.001**
**Lymphocyte count (cells/L)**	1.89 (0.68)	2.02 (0.66)	1.86 (0.68)	1.88 (0.68)	**<0.001**
**Lymphocyte percentage (%)**	31.7 (10.0)	33.0 (9.99)	33.0 (9.80)	29.7 (10.1)	**<0.001**
**Monocyte count (cells/L)**	0.41 (0.16)	0.43 (0.16)	0.39 (0.15)	0.43 (0.17)	**<0.001**
**Monocyte percentage (%)**	6.64 (1.92)	6.91 (1.93)	6.71 (1.91)	6.47 (1.90)	**<0.001**
**Neutrophil count (cells/L)**	3.78 (1.87)	3.77 (1.80)	3.44 (1.62)	4.24 (2.08)	**<0.001**
**Neutrophil percentage (%)**	58.80 (11.20)	57.10 (11.00)	57.40 (10.80)	61.30 (11.30)	**<0.001**
**Platelet count (cells/L)**	208.00 (66.60)	214.00 (66.40)	205.00 (66.40)	209.00 (66.80)	**<0.001**
**White blood cell leukocyte count (cells/L)**	6.25 (2.25)	6.40 (2.03)	5.86 (2.23)	6.71 (2.27)	**<0.001**
**NLR**	2.32 (2.00)	2.12 (1.79)	2.13 (1.86)	2.63 (2.20)	**<0.001**
**MLR**	0.24 (0.13)	0.23 (0.11)	0.23 (0.12)	0.25 (0.14)	**<0.001**
**PLR**	121.00 (59.80)	116.00 (54.80)	120.00 (58.90)	123.00 (62.30)	**<0.001**
**Hematocrit percentage (%)**	41.00 (23.00)	41.00 (5.00)	41.00 (5.00)	0.42 (0.36)	**<0.001**
**Red blood cell count (cells/L)**	4.54 (0.57)	4.56 (0.54)	4.47 (0.57)	4.63 (0.58)	**<0.001**
**Mean corpuscular hemoglobin concentration (g/dL)**	328.00 (13.70)	328.00 (11.80)	327.00 (15.10)	329.00 (12.30)	**<0.001**
**Mean corpuscular hemoglobin (pg)**	29.90 (2.41)	29.80 (2.49)	29.90 (2.49)	29.80 (2.29)	**0.013**
**Mean corpuscular volume (fL)**	90.90 (6.20)	90.70 (6.38)	91.20 (6.35)	90.70 (5.93)	**<0.001**
**Direct LDL (mmol/L)**	2.30 (0.74)	2.24 (0.71)	2.40 (0.75)	2.20 (0.72)	**<0.001**
**Triglycerides (mmol/L)**	1.22 (0.89)	1.20 (0.89)	1.25 (0.88)	1.19 (0.90)	**<0.001**
**Total cholesterol (mmol/L)**	4.12 (0.92)	4.04 (0.89)	4.23 (0.94)	3.99 (0.89)	**<0.001**
**Albumin (g/L)**	43.50 (4.07)	43.90 (3.89)	42.80 (3.84)	44.20 (4.27)	**<0.001**
**Alanine aminotransferase (U/L)**	22.80 (26.20)	21.50 (22.50)	21.40 (26.00)	25.00 (27.40)	**<0.001**
**Glutamyltransferase (U/L)**	23.30 (39.70)	22.50 (40.80)	23.90 (42.90)	22.90 (34.50)	0.093
**Aspartate aminotransferase (U/L)**	22.40 (20.90)	21.90 (15.50)	21.70 (23.20)	23.40 (19.00)	**<0.001**
**Direct bilirubin (µmol/L)**	3.92 (4.81)	3.63 (5.48)	3.69 (4.18)	4.31 (5.30)	**<0.001**
**Total bilirubin ( µmol/L)**	11.00 (7.59)	10.10 (8.24)	10.80 (6.85)	11.50 (8.23)	**<0.001**
**Creatinine (µmol/L)**	65.50 (22.30)	65.50 (17.60)	64.40 (23.80)	67.00 (21.60)	**<0.001**
**Cystatin C (mg/L)**	0.85 (0.19)	0.83 (0.14)	0.85 (0.20)	0.85 (0.20)	**<0.001**
**Total protein (g/L)**	68.50 (5.63)	68.50 (5.37)	67.70 (5.33)	69.50 (5.95)	**<0.001**
**Urate (µmol/L)**	313 (93.10)	324 (91.90)	296 (85.50)	332.00 (98.80)	**<0.001**
**Urea (mmol/L)**	4.59 (1.57)	4.49 (1.49)	4.60 (1.59)	4.61 (1.56)	**<0.001**
**Calcium (mmol/L)**	2.27 (0.12)	2.28 (0.11)	2.26 (0.11)	2.28 (0.12)	**<0.001**
**Alkaline phosphatase (U/L)**	80.80 (42.30)	79.70 (40.90)	78.70 (40.10)	83.90 (45.30)	**<0.001**
**Glucose (mmol/L)**	4.97 (1.01)	4.91 (0.99)	4.86 (0.89)	5.13 (1.15)	**<0.001**

The bold values indicate that the p-value was statistically significant.

**Figure 3:**
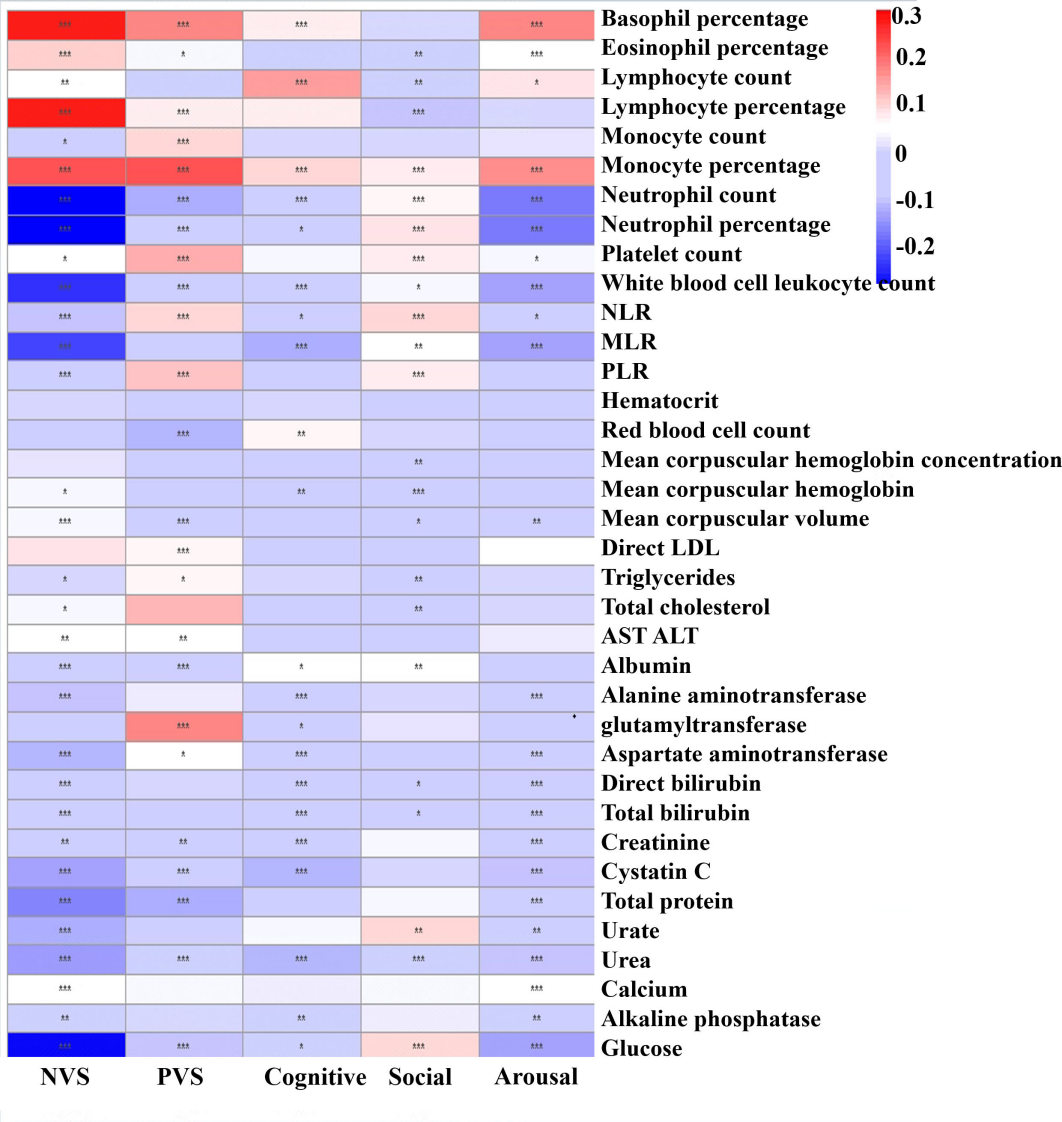
Heatmap showing adjusted effect sizes (*ß*) for associations between peripheral blood test index with RDoC domain scores in the total population. The color reflects the effect size: red means a high positive effect and blue means negative effect. *p < 0.05, **p < 0.01, ***p < 0.001.

**Figure 3** presents adjusted associations between each RDoC score and a series of blood test indexes. Those seen to be more related to the NVS included basophil percentage (adjusted *β* = 0.275, *p* < 0.001), lymphocyte percentage (adjusted *β* = 0.271, *p* < 0.001), monocyte percentage (adjusted *β* = 0.223, *p* < 0.001), neutrophil percentage (adjusted *β* = −0.310, *p* < 0.001), neutrophil count (adjusted *β* = −0.301, *p* < 0.001), glucose (adjusted *β* = −0.287, *p* < 0.001), white blood cell leukocyte count (adjusted *β* = −0.244, *p* < 0.001), NLR (adjusted *β* = −0.229, *p* < 0.001), and total protein (adjusted *β* = −0.170, *p* < 0.001). More relevant to PVS included monocyte percentage (adjusted *β* = 0.228, *p* < 0.001), basophil count (adjusted *β* = 0.176, *p* < 0.001), and glutamyl transferase (adjusted *β* = 0.171, *p* < 0.001). Those more related to the cognitive system domain included lymphocyte count (adjusted *β* = 0.150, *p* < 0.001). More relevant to the social process domain was lymphocyte percentage (adjusted *β* = −0.102, *p* < 0.001). More relevant to the arousal domain were basophil percentage (adjusted *β* = 0.180, *p* < 0.001), monocyte percentage (adjusted *β* = 0.168, *p* < 0.001), neutrophil percentage (adjusted *β* = −0.178, *p* < 0.001), and neutrophil count (adjusted *β* = −0173, *p* < 0.001). Overall, it is evident that monocyte percentage and lymphocyte percentage as inflammatory factors might be significance indicators with various RDoC domain scores. The association results in MDD, BD, and SCZ populations are shown in **Supplementary Figures S1**-**S3**; however, the results were slightly heterogeneous from the total population, suggesting that this association was not so robust.

**Figure 4:**
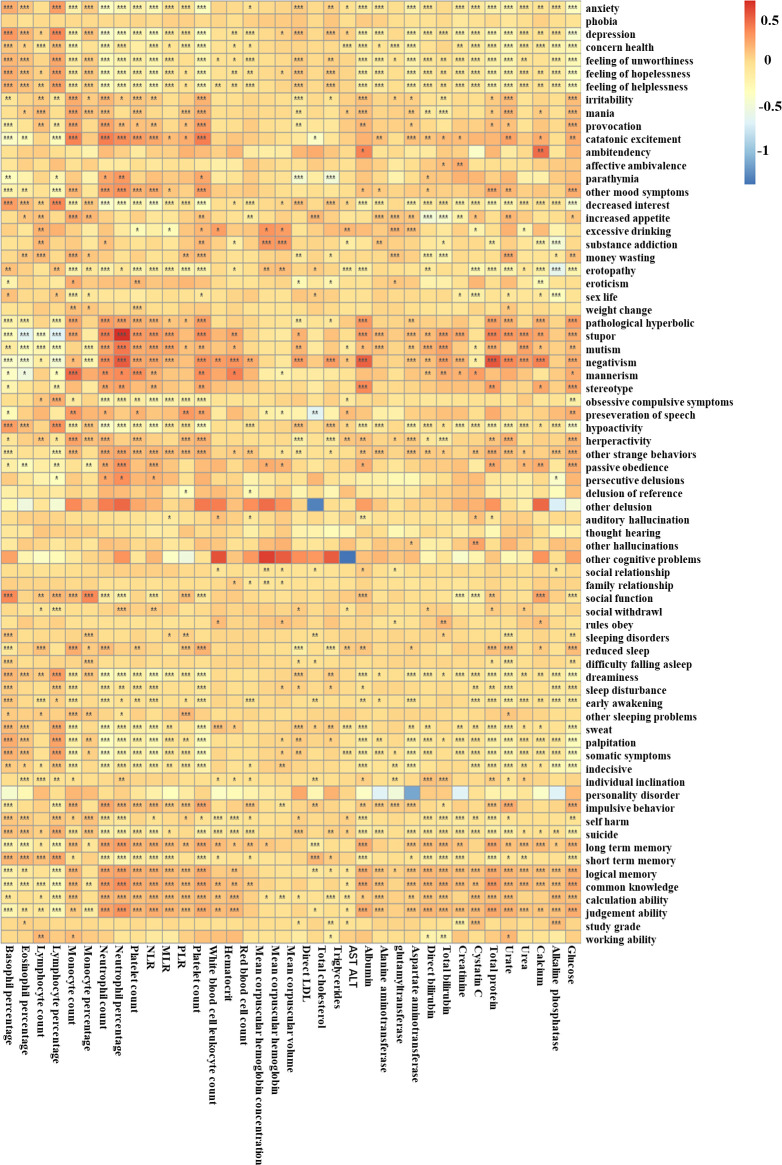
The associations between peripheral blood test indexes with different symptoms in the total population. The color reflects the effect size: red means a high positive effect and blue means a negative effect. *p < 0.05, **p < 0.01, ***p < 0.001.

**Figure 4** shows the heatmap of associations between symptoms and blood test using the minimally adjusted logit regression model. For mood-related symptoms, negative symptoms such as *anxiety*, *depression*, *feeling of worthlessness*, *feeling of hopelessness*, and *feeling of helplessness* were more strongly correlated with higher basophil percentage (adjusted *β* = 0.220–0.349, *p* < 0.001), lower glucose (adjusted *β* = −0.443 to −0.296, *p* < 0.001), lower white blood cell count (adjusted *β* = −0.367 to −0.237, *p* < 0.001), lower neutrophil counts (adjusted *β* = −0.423 to −0.284, *p* < 0.001), and lower neutrophil percentage (adjusted *β* = −0.368 to −0.237, *p* < 0.001). The symptom *depression* also showed stronger correlations with higher eosinophil percentage (adjusted *β* = 0.148, *p* < 0.001), lower monocyte percentage (adjusted *β* = −0.195, *p* < 0.001), and higher direct LDL (adjusted *β* = 0.111, *p* < 0.001) than *anxiety* (adjusted *β* = −0.03–0.053, *p* > 0.500), while “positive” symptoms such as *irritability*, *mania*, *provocation*, and *catatonic excitement* were associated with higher monocyte count (adjusted *β* = 0.191–0.362, *p* < 0.001), higher white blood cell count (adjusted *β* = 0.171–0.389, *p* < 0.001), and higher neutrophil count (adjusted *β* = 0.118–0.375, *p* < 0.001). In this respect, it is more similar to “positive” symptoms. Reward-related symptoms including *increased appetite*, *eroticism*, and *money wasting* were associated with higher monocyte count (adjusted *β* = 0.169–0.210, *p* < 0.001). For cognitive-related symptoms including *auditory hallucinations* and *other hallucinations*, they were associated with higher cystatin C (adjusted *β* = 0.116–0.239, *p* < 0.01). For arousal-related symptoms, *dreaminess*, *sweat*, *palpitation*, and *somatic symptoms* were associated with higher lymphocyte percentage (*β* = 0.194–0.253, *p* < 0.001), higher basophil percentage (adjusted *β* = 0.124–0.189, *p* < 0.001), lower albumin (adjusted *β* = −0.158 to −0.118, *p* < 0.001), lower total protein (adjusted *β* = −0.189 to −0.147, *p* < 0.01), lower urate (adjusted *β* = −0.235 to −0.143, *p* < 0.001), lower white blood cell count (adjusted *β* = −0.3454 to −0.2421, *p* < 0.001), lower glucose (adjusted *β* = −0.334 to −0.224, *p* < 0.001), lower neutrophil percentage (adjusted *β* = −0.269 to −0.197, *p* < 0.001), and lower neutrophil count (adjusted *β* = −0.378 to −0.275, *p* < 0.001). Sensorimotor symptoms such as *stupor*, *mutism*, *negativism*, *mannerism*, and *stereotype* were associated with higher white blood cell count (adjusted *β* = 0.148–0.331, *p* < 0.001), higher neutrophil percentage (adjusted *β* = 0.244–0.694, *p* < 0.006), higher neutrophil count (adjusted *β* = 0.206–0.390, *p* < 0.004), and lower basophil percentage (adjusted *β* = −0.420 to −0.156, *p* < 0.001). For cognitive ability, poorer cognitive ability including *long-term memory*, *logical memory*, *common knowledge*, *calculation ability*, and *judgment ability* were associated with higher neutrophil percentage (adjusted *β* = 0.160–0.319, *p* < 0.001), total protein (adjusted *β* = 0.109–0.278, *p* < 0.001), higher neutrophil count (adjusted *β* = 0.146–0.255, *p* < 0.001), higher white blood cell count (adjusted *β* = 0.124–0.218, *p* < 0.001), and higher glucose (adjusted *β* = 0.107–0.212, *p* < 0.001). *Short-term memory*, on the other hand, presented opposite relationships with the same indexes from other cognitive abilities (adjusted *β* = −0.225 to −0.112, *p* < 0.001), suggesting a possible opposite pathophysiological mechanism behind them. The results for the MDD, BD, and SCZ populations are shown in **Supplementary Figures S4**-**S6**, also slightly heterogeneous from the total population. It should be noted that considerable relationships were detected between hematocrit and multiple symptoms in the SCZ population, suggesting that hematocrit might play a considerable role in multiple symptoms in the SCZ population.

## Discussion

In our study, we demonstrated a large number of blood features associated with specific DSM-5 diagnosis, RDoC characteristics, and various precise symptoms, and proved the heterogeneity of psychiatric disorders in the presentation of peripheral blood indexes to some extent. Overall, in the SCZ population, a higher proportion of abnormal inflammatory index is suggested. This illustrates the possible relationship between SCZ and higher inflammation, consistent with other studies (35, 36). The link between SCZ and the immune system has been proposed, including associations with immune loci (37) and genetic overlap with immune-mediated diseases (38), though the exact immune mechanisms of SCZ remain unknown. It has also been noted that genetic factors involving leukocyte counts were associated with the risk of SCZ (39).

The associations between RDoC domain scores and peripheral blood indexes showed various degrees of associations between NVS, PVS, and a series of inflammatory factors such as eosinophils and lymphocytes. These results were consistent with other findings on NVS and PVS domains including stress (40). A series of studies suggested that inflammation could enhance the processing of aversive stimuli, including interoceptive signals, and activate emotional arousal networks (41–43). Several lines of evidence indicated that inflammation could also foster the formation and persistence of interoceptive fear memories (44). Additionally, glucose, urea, urate, cystatin C, albumin, and calcium showed statistically significant associations with five RDoC domains, but weaker compared to the associations of inflammatory factors with both NVS and PVS. Such associations are also reflected in the RDoC matrix, such as the construct of loss associated with inflammatory molecules.

Overall, the inflammatory-related indexes were associated with almost all recorded symptoms, especially mood-related and reward-related symptoms, which is consistent with the findings of other studies (45–48). Seaton et al. (49) noted that changes in inflammatory factors could be altered by intervening on mood. Pike et al. (47) found that dynamic hyperreactivity of inflammatory cells, such as peripheral blood leukocytes, was associated with blunted activation of the reward system and lower subjective pleasure expectancy in MDD patients. The results suggested the inflammation–brain–behavior hypothesis, particularly highlighted on the interventions of inflammatory-related factors in patients with mood and reward symptoms. In terms of mood-related symptoms, “positive” symptoms like *mania* and *irritability* and “negative” symptoms like *depression* and *anxiety* showed considerable associations with the same blood indexes, but showed almost opposite correlations, suggesting the possible two ends of one psychopathology continuum.

Surprisingly, cystatin C has been found to be associated with *hallucinations*. As few studies have found this association in MDD, BD, or SCZ, cystatin C appeared to be involved in amyloid-like brain dysfunction such as Alzheimer’s disease (50), cognitive disorders (51), and epilepsy (52). Moreover, multiple *in vitro* and *in vivo* findings have demonstrated that cystatin C induces cellular autophagy and cellular proliferation, or inhibits amyloid-β (Aβ) aggregation through pathways dependent on the inhibition of endosomal–lysosomal pathway cysteine proteases, such as tissue protease B.

For arousal-related symptoms, inflammatory factors including lymphocyte percentage and white blood cell count also played a considerable role. Sleep has been identified to play a dynamic role in regulating the HPA axis as well as the immune system (53, 54), and sleep is thought to provide a survival advantage supporting a neurally integrated immune system that might anticipate injury and infectious threats (55). On the other hand, inflammatory signals are conveyed to the CNS via neural innervation and the actions of humoral mediators, resulting in poor sleep (56, 57). Moreover, albumin and urate showed associations with a large number of symptoms. Several studies have confirmed a more pronounced relationship between albumin and sleep duration, depressive symptoms, etc. (58, 59). The mechanism might be that depression might involve inflammatory, immune, oxidative, and nitrosative stress with microprogrammed expression responses (60, 61), and albumin played an essential role in this process. Urate has also been detected in Parkinson in relation to sleep (62) and suspected to be involved in the disease pathogenesis of synucleinopathies in neurodegenerative diseases.

Regarding sensorimotor symptoms, associations between *stupor*, *negativism*, *mannerism*, and a series of inflammatory factors, albumin, and total protein have been demonstrated. Some studies have shown that *catatonia* including *stupor* seems to be less likely to be caused by systemic inflammation (63, 64) and inflammation played a role in *mannerism* (65). Rogers et al. revealed that N-methyl-D-aspartate receptor (NMDAR) encephalitis could account for the full spectrum of catatonic features including *stupor*. Other studies have pointed to the role of the liver in symptoms such as *stupor* in hepatic encephalopathy (66).

The associations between cognitive abilities and blood index, despite strong, were almost all not statistically significant. In terms of inflammatory factors, several studies have examined the relationship between inflammatory cytokines and cognitive performance, highlighting the significance of IL-8, IL-6, and CRP (67–69). The interaction between neurodevelopment and inflammation was thought to play a considerably important role (70). On the other hand, cognitive abilities were demonstrated to statistically significantly associate with total protein and glucose. Among these, total protein has been proposed with increased immunoglobulins, but there are no studies that examine its relationship with psychiatric disorders. Moreover, some studies have found a more pronounced relationship between cognitive decline and glucose in patients with SCZ (71), and the interaction between abnormal glucose metabolism and white matter connectivity disorders in the SCZ population might lead to cognitive impairment (72).

In addition, in the SCZ population, hematocrit has been found to be associated with multiple symptoms, suggesting the significance of its role in neuro-related disease. However, the hematocrit was thought to be significant in anxiety and depression (73, 74). Thus, the notable associations with various symptoms in SCZ could be explored in the near future.

Surprisingly, similarities in association with the same blood test indexes have been found in similar symptoms in terms of RDoC thinking. Because RDoC classification was based on a clear neuroscientific basis, our results illustrated the validity of this classification thinking to some extent. Similarly, the opposite relationships of blood test indexes between “positive” and “negative” moods were found, suggesting that there might be a continuum spectrum of low–high symptoms with a certain pathology.

## Conclusion

The proportion of high inflammatory indexes in SCZ was relatively high, but in terms of mean values, SCZ, BD, and MDD did not differ significantly. Inflammatory response showed a strong correlation with NVS, PVS, and a range of psychiatric symptoms especially mood symptoms, psychomotor symptoms, and cognitive abilities. Albumin, total protein, and uric acid also showed strong associations. Similar symptoms clustered according to RDoC thinking had similar associations with certain blood markers, while “positive” and “negative” symptoms had opposite relationships, suggesting that these symptoms might be on a continuous spectrum.

## Limitation

Several limitations must be mentioned. First, as our study was a cross-sectional study, it is not possible to distinguish specific causal relationships. Second, symptoms were obtained by doctors through interviews, and no scale was used. Moreover, the free text information collected on the symptoms was only used to form a dichotomous category of “present/absent” or “good/poor” through regular expressions. Furthermore, a large number of blood markers were not included in this study due to the high proportion of missing values, including IL-6 and CRP. In addition, our current study assumes that there is a generalized linear relationships between disease, symptoms, and peripheral blood markers, but more complex relationships such as U-traits might exist. Because our study was designed to explore as many associations as possible, more complex association analysis was not performed. In addition, we did not consider more possible environmentally relevant confounders, and healthy subjects were not included in our study. Therefore, there are limitations within the generalizability of the present results.

## Data Availability

The raw data supporting the conclusions of this article will be made available by the authors, without undue reservation.
