# Supplementary material for: Association between psychiatric symptoms with multiple peripheral blood sample test: a 10-year retrospective study
Source: Front Psychiatry. 2024 Dec 9;15:1481006. doi: 10.3389/fpsyt.2024.1481006 (PMC11663843; doi:10.3389/fpsyt.2024.1481006)
Supplement: Supplementary file 3 [file DataSheet3.docx]

**Table S1 The symptom**

| Mood symptom | anxiety  depression  phobia  concern health  feeling of unworthiness  feeling of hopelessness  feeling of helplessness  irritability  mania  provocation  catatonic excitement  ambitendency  affective ambivalence  parathymia  other mood symptoms |
| --- | --- |
| Reward symptom | decreased interest  increased appetite  excessive drinking  substance addiction  money wasting  erotopathy  eroticism  sex life  weight change |
| Seniormotor | pathological hyperbolic  stupor  mutism  negativism  mannerism  stereotype  obsessive compulsive symptoms  preseveration of speech  hypoactivity  herperactivity  other strange behaviors |
| Cognitive symptoms | passive obedience  persecutive delusions  delusion of reference  other delusion  auditory hallucination  thought hearing  other hallucinations  other cognitive problems |
| Social problems | social relationship  social function  social withdrawl  rules obey  family relationship |
| Arousal problems | sleeping problem  reduced sleep  difficulty falling asleep  dreaminess  sleep disturbance  early awakening  other sleeping problems  sweat  palpitation  somatic symptoms |
| Personality | indecisive  individual inclination  personality disorder |
| Harm behavior | self harm  suicide  impulsive behavior |
| Cognitive ability | long term memory  short term memory  logical memory  common knowledge  calculation ability  judgement ability  study grade  working ability |

Table S2 Peripheral blood test

| inflammatory related | Basophil percentage  Eosinophil percentage  Lymphocyte count  Lymphocyte percentage  Monocyte count  Monocyte percentage  Neutrophil count  Neutrophil percentage  Platelet count  White blood cell leukocyte count  NLR  MLR  PLR |
| --- | --- |
| hemotological related | Hematocrit  Red blood cell count  Mean corpuscular hemoglobin concentration  Mean corpuscular hemoglobin  Mean corpuscular volume |
| cardiovascular related | Direct LDL  Triglycerides  Total cholesterol |
| liver related | Albumin  Alanine aminotransferase  glutamyltransferase  Aspartate aminotransferase  Direct bilirubin  Total bilirubin |
| renal related | Creatinine  Cystatin C  Total protein  Urate  Urea |
| bone related | Calcium  Alkaline phosphatase |
| DM related | Glucose |

**Table S3 The characteristics of symptoms in various DSM-5 categories**

| Covariate | Full Sample (n=18903) | BD (n=2344) | MDD (n=8198) | SCZ (n=8361) | p-value |
| --- | --- | --- | --- | --- | --- |
| **anxiety** |  |  |  |  | $<$**0.001** |
| no | 11540 (62) | 1541 (66) | 2779 (34) | 7220 (89) |  |
| yes | 7078 (38) | 787 (34) | 5383 (66) | 908 (11) |  |
| Missing | **285** | **16** | **36** | **233** |  |
| **phobia** |  |  |  |  | 0.12 |
| no | 18076 (97) | 2265 (97) | 7902 (97) | 7909 (97) |  |
| yes | 566 (3) | 63 (3) | 272 (3) | 231 (3) |  |
| Missing | **261** | **16** | **24** | **221** |  |
| **depression** |  |  |  |  | $<$**0.001** |
| no | 8038 (43) | 949 (41) | 606 (7) | 6483 (80) |  |
| yes | 10552 (57) | 1376 (59) | 7554 (93) | 1622 (20) |  |
| Missing | **313** | **19** | **38** | **256** |  |
| **irritability** |  |  |  |  | $<$**0.001** |
| no | 17382 (92) | 1498 (64) | 8000 (98) | 7884 (94) |  |
| yes | 1510 (8) | 845 (36) | 194 (2) | 471 (6) |  |
| Missing | **11** | **1** | **4** | **6** |  |
| **mania** |  |  |  |  | $<$**0.001** |
| no | 17219 (92) | 1361 (58) | 8040 (98) | 7818 (96) |  |
| yes | 1427 (8) | 969 (42) | 131 (2) | 327 (4) |  |
| Missing | **257** | **14** | **27** | **216** |  |
| **provocation** |  |  |  |  | $<$**0.001** |
| no | 18468 (98) | 2050 (87) | 8165 (100) | 8253 (99) |  |
| yes | 424 (2) | 293 (13) | 29 (0) | 102 (1) |  |
| Missing | **11** | **1** | **4** | **6** |  |
| **catatonic excitement** |  |  |  |  | $<$**0.001** |
| no | 18677 (99) | 2316 (99) | 8168 (100) | 8193 (99) |  |
| yes | 135 (1) | 21 (1) | 16 (0) | 98 (1) |  |
| Missing | **91** | **7** | **14** | **70** |  |
| **ambitendency** |  |  |  |  | **0.047** |
| no | 18778 (100) | 2335 (100) | 8176 (100) | 8267 (100) |  |
| yes | 32 (0) | 2 (0) | 9 (0) | 21 (0) |  |
| Missing | **93** | **7** | **13** | **73** |  |
| **affective ambivalence** |  |  |  |  | **0.011** |
| no | 18574 (100) | 2315 (99) | 8147 (100) | 8112 (99) |  |
| yes | 91 (0) | 18 (1) | 27 (0) | 46 (1) |  |
| Missing | **238** | **11** | **24** | **203** |  |
| **parathymia** |  |  |  |  | $<$**0.001** |
| no | 18582 (100) | 2325 (100) | 8169 (100) | 8088 (99) |  |
| yes | 83 (0) | 7 (0) | 6 (0) | 70 (1) |  |
| Missing | **238** | **12** | **23** | **203** |  |
| **feeling of unworthiness** |  |  |  |  | $<$**0.001** |
| no | 13659 (72) | 1603 (68) | 4213 (51) | 7843 (94) |  |
| yes | 5233 (28) | 740 (32) | 3982 (49) | 511 (6) |  |
| Missing | **11** | **1** | **3** | **7** |  |
| **feeling of hopelessness** |  |  |  |  | $<$**0.001** |
| no | 12269 (65) | 1455 (62) | 3155 (38) | 7659 (92) |  |
| yes | 6623 (35) | 888 (38) | 5040 (62) | 695 (8) |  |
| Missing | **11** | **1** | **3** | **7** |  |
| **feeling of helplessness** |  |  |  |  | $<$**0.001** |
| no | 12906 (68) | 1532 (65) | 3630 (44) | 7744 (93) |  |
| yes | 5986 (32) | 811 (35) | 4565 (56) | 610 (7) |  |
| Missing | **11** | **1** | **3** | **7** |  |
| **other mood symptoms** |  |  |  |  | $<$**0.001** |
| no | 18179 (97) | 2312 (99) | 8140 (100) | 7727 (95) |  |
| yes | 494 (3) | 21 (1) | 35 (0) | 438 (5) |  |
| Missing | **230** | **11** | **23** | **196** |  |
| **decreased interest** |  |  |  |  | $<$**0.001** |
| no | 9384 (50) | 1111 (48) | 1234 (15) | 7039 (85) |  |
| yes | 9367 (50) | 1220 (52) | 6946 (85) | 1201 (15) |  |
| Missing | **152** | **13** | **18** | **121** |  |
| **increased appetite** |  |  |  |  | $<$**0.001** |
| no | 18461 (98) | 2056 (88) | 8144 (99) | 8261 (99) |  |
| yes | 431 (2) | 287 (12) | 50 (1) | 94 (1) |  |
| Missing | **11** | **1** | **4** | **6** |  |
| **alcohol history** |  |  |  |  | $<$**0.001** |
| good | 1927 (10) | 314 (13) | 1003 (12) | 610 (7) |  |
| poor | 16975 (90) | 2030 (87) | 7195 (88) | 7750 (93) |  |
| Missing | **1** | **0** | **0** | **1** |  |
| **excessive drinking** |  |  |  |  | $<$**0.001** |
| no | 18700 (99) | 2287 (98) | 8139 (99) | 8274 (100) |  |
| yes | 114 (1) | 49 (2) | 45 (1) | 20 (0) |  |
| Missing | **89** | **8** | **14** | **67** |  |
| **substance addiction** |  |  |  |  | $<$**0.001** |
| no | 18557 (98) | 2296 (98) | 8100 (99) | 8161 (98) |  |
| yes | 331 (2) | 43 (2) | 93 (1) | 195 (2) |  |
| Missing | **15** | **5** | **5** | **5** |  |
| **drug history** |  |  |  |  | 0.97 |
| no | 18860 (100) | 2339 (100) | 8178 (100) | 8343 (100) |  |
| yes | 42 (0) | 5 (0) | 19 (0) | 18 (0) |  |
| Missing | **1** | **0** | **1** | **0** |  |
| **money wasting** |  |  |  |  | $<$**0.001** |
| no | 18247 (97) | 1863 (80) | 8156 (100) | 8228 (98) |  |
| yes | 645 (3) | 480 (20) | 38 (0) | 127 (2) |  |
| Missing | **11** | **1** | **4** | **6** |  |
| **erotopathy** |  |  |  |  | $<$**0.001** |
| no | 18082 (96) | 2241 (96) | 7538 (92) | 8303 (99) |  |
| yes | 807 (4) | 102 (4) | 654 (8) | 51 (1) |  |
| Missing | **14** | **1** | **6** | **7** |  |
| **eroticism** |  |  |  |  | $<$**0.001** |
| no | 18782 (99) | 2267 (97) | 8191 (100) | 8324 (100) |  |
| yes | 110 (1) | 76 (3) | 3 (0) | 31 (0) |  |
| Missing | **11** | **1** | **4** | **6** |  |
| **sex life** |  |  |  |  | $<$**0.001** |
| good | 17662 (97) | 2177 (97) | 7616 (96) | 7869 (98) |  |
| poor | 556 (3) | 59 (3) | 318 (4) | 179 (2) |  |
| Missing | **685** | **108** | **264** | **313** |  |
| **weight change** |  |  |  |  | $<$**0.001** |
| no | 18207 (97) | 2134 (91) | 7949 (97) | 8124 (98) |  |
| yes | 617 (3) | 206 (9) | 233 (3) | 178 (2) |  |
| Missing | **79** | **4** | **16** | **59** |  |
| **concern health** |  |  |  |  | $<$**0.001** |
| no | 16296 (86) | 2090 (89) | 6033 (74) | 8173 (98) |  |
| yes | 2596 (14) | 253 (11) | 2162 (26) | 181 (2) |  |
| Missing | **11** | **1** | **3** | **7** |  |
| **pathological hyperbolic** |  |  |  |  | $<$**0.001** |
| no | 18282 (97) | 2190 (94) | 8142 (99) | 7950 (96) |  |
| yes | 507 (3) | 146 (6) | 41 (1) | 320 (4) |  |
| Missing | **114** | **8** | **15** | **91** |  |
| **stupor** |  |  |  |  | $<$**0.001** |
| no | 18622 (99) | 2328 (100) | 8149 (100) | 8145 (98) |  |
| yes | 193 (1) | 9 (0) | 35 (0) | 149 (2) |  |
| Missing | **88** | **7** | **14** | **67** |  |
| **obsessive compulsive symptoms** |  |  |  |  | $<$**0.001** |
| no | 18157 (97) | 2249 (96) | 7898 (97) | 8010 (98) |  |
| yes | 566 (3) | 86 (4) | 278 (3) | 202 (2) |  |
| Missing | **180** | **9** | **22** | **149** |  |
| **preseveration of speech** |  |  |  |  | $<$**0.001** |
| no | 18728 (100) | 2326 (100) | 8174 (100) | 8228 (99) |  |
| yes | 84 (0) | 11 (0) | 10 (0) | 63 (1) |  |
| Missing | **91** | **7** | **14** | **70** |  |
| **hypoactivity** |  |  |  |  | $<$**0.001** |
| no | 9804 (52) | 1192 (51) | 1610 (20) | 7002 (85) |  |
| yes | 8958 (48) | 1143 (49) | 6570 (80) | 1245 (15) |  |
| Missing | **141** | **9** | **18** | **114** |  |
| **herperactivity** |  |  |  |  | $<$**0.001** |
| no | 17728 (94) | 1485 (63) | 8136 (99) | 8107 (97) |  |
| yes | 1164 (6) | 858 (37) | 58 (1) | 248 (3) |  |
| Missing | **11** | **1** | **4** | **6** |  |
| **other strange behaviors** |  |  |  |  | $<$**0.001** |
| no | 17363 (92) | 2263 (97) | 8113 (99) | 6987 (84) |  |
| yes | 1461 (8) | 74 (3) | 72 (1) | 1315 (16) |  |
| Missing | **79** | **7** | **13** | **59** |  |
| **passive obedience** |  |  |  |  | $<$**0.001** |
| no | 18629 (99) | 2327 (100) | 8150 (100) | 8152 (98) |  |
| yes | 187 (1) | 10 (0) | 35 (0) | 142 (2) |  |
| Missing | **87** | **7** | **13** | **67** |  |
| **persecutive delusions** |  |  |  |  | **0.0019** |
| no | 18844 (100) | 2339 (100) | 8183 (100) | 8322 (100) |  |
| yes | 58 (0) | 4 (0) | 15 (0) | 39 (0) |  |
| Missing | **1** | **1** | **0** | **0** |  |
| **delusions of reference** |  |  |  |  | **0.021** |
| no | 18846 (100) | 2339 (100) | 8181 (100) | 8326 (100) |  |
| yes | 56 (0) | 4 (0) | 17 (0) | 35 (0) |  |
| Missing | **1** | **1** | **0** | **0** |  |
| **other delusions** |  |  |  |  | **0.032** |
| no | 18894 (100) | 2342 (100) | 8198 (100) | 8354 (100) |  |
| yes | 8 (0) | 1 (0) | 0 (0) | 7 (0) |  |
| Missing | **1** | **1** | **0** | **0** |  |
| **auditory hallucinations** |  |  |  |  | $<$**0.001** |
| no | 18309 (97) | 2298 (98) | 8092 (99) | 7919 (95) |  |
| yes | 561 (3) | 46 (2) | 104 (1) | 411 (5) |  |
| Missing | **33** | **0** | **2** | **31** |  |
| **thought hearing** |  |  |  |  | $<$**0.001** |
| no | 18823 (100) | 2340 (100) | 8193 (100) | 8290 (100) |  |
| yes | 46 (0) | 4 (0) | 2 (0) | 40 (0) |  |
| Missing | **34** | **0** | **3** | **31** |  |
| **other hallucinations** |  |  |  |  | $<$**0.001** |
| no | 18744 (99) | 2332 (99) | 8168 (100) | 8244 (99) |  |
| yes | 127 (1) | 12 (1) | 28 (0) | 87 (1) |  |
| Missing | **32** | **0** | **2** | **30** |  |
| **other cognitive problems** |  |  |  |  | **0.043** |
| no | 18897 (100) | 2343 (100) | 8198 (100) | 8356 (100) |  |
| yes | 5 (0) | 0 (0) | 0 (0) | 5 (0) |  |
| Missing | **1** | **1** | **0** | **0** |  |
| **language organization** |  |  |  |  | $<$**0.001** |
| no | 18848 (100) | 2340 (100) | 8186 (100) | 8322 (100) |  |
| yes | 53 (0) | 3 (0) | 12 (0) | 38 (0) |  |
| Missing | **2** | **1** | **0** | **1** |  |
| **long term memory** |  |  |  |  | $<$**0.001** |
| good | 17746 (95) | 2261 (97) | 7936 (97) | 7549 (91) |  |
| poor | 1020 (5) | 69 (3) | 241 (3) | 710 (9) |  |
| Missing | **137** | **14** | **21** | **102** |  |
| **short term memory** |  |  |  |  | $<$**0.001** |
| no | 1294 (7) | 118 (5) | 382 (5) | 794 (10) |  |
| yes | 17476 (93) | 2212 (95) | 7796 (95) | 7468 (90) |  |
| Missing | **133** | **14** | **20** | **99** |  |
| **logical memory** |  |  |  |  | $<$**0.001** |
| good | 17408 (93) | 2225 (96) | 7982 (98) | 7201 (87) |  |
| poor | 1351 (7) | 104 (4) | 193 (2) | 1054 (13) |  |
| Missing | **144** | **15** | **23** | **106** |  |
| **common knowledge** |  |  |  |  | $<$**0.001** |
| good | 18038 (95) | 2293 (98) | 8057 (98) | 7688 (92) |  |
| poor | 865 (5) | 51 (2) | 141 (2) | 673 (8) |  |
| **calculation ability** |  |  |  |  | $<$**0.001** |
| good | 16884 (89) | 2174 (93) | 7588 (93) | 7122 (85) |  |
| poor | 2019 (11) | 170 (7) | 610 (7) | 1239 (15) |  |
| **judgement ability** |  |  |  |  | $<$**0.001** |
| good | 17555 (94) | 2250 (97) | 7996 (98) | 7309 (89) |  |
| poor | 1205 (6) | 79 (3) | 179 (2) | 947 (11) |  |
| Missing | **143** | **15** | **23** | **105** |  |
| **study grade** |  |  |  |  | $<$**0.001** |
| not poor | 17429 (92) | 2201 (94) | 7765 (95) | 7463 (89) |  |
| poor | 1441 (8) | 140 (6) | 417 (5) | 884 (11) |  |
| Missing | **33** | **3** | **16** | **14** |  |
| **working ability** |  |  |  |  | $<$**0.001** |
| good | 18597 (99) | 2178 (93) | 8161 (100) | 8258 (100) |  |
| poor | 209 (1) | 157 (7) | 19 (0) | 33 (0) |  |
| Missing | **97** | **9** | **18** | **70** |  |
| **working well** |  |  |  |  | $<$**0.001** |
| no | 1145 (6) | 122 (5) | 363 (4) | 660 (8) |  |
| yes | 17699 (94) | 2214 (95) | 7810 (96) | 7675 (92) |  |
| Missing | **59** | **8** | **25** | **26** |  |
| **social relationship** |  |  |  |  | $<$**0.001** |
| good | 17359 (92) | 2167 (93) | 7743 (95) | 7449 (89) |  |
| poor | 1493 (8) | 169 (7) | 432 (5) | 892 (11) |  |
| Missing | **51** | **8** | **23** | **20** |  |
| **social function** |  |  |  |  | $<$**0.001** |
| no | 1246 (7) | 131 (6) | 466 (6) | 649 (8) |  |
| yes | 17653 (93) | 2213 (94) | 7730 (94) | 7710 (92) |  |
| Missing | **4** | **0** | **2** | **2** |  |
| **social withdrawl** |  |  |  |  | $<$**0.001** |
| no | 16939 (90) | 2227 (95) | 7589 (93) | 7123 (86) |  |
| yes | 1872 (10) | 110 (5) | 594 (7) | 1168 (14) |  |
| Missing | **92** | **7** | **15** | **70** |  |
| **rules obey** |  |  |  |  | $<$**0.001** |
| no | 397 (2) | 51 (2) | 80 (1) | 266 (3) |  |
| yes | 18452 (98) | 2285 (98) | 8095 (99) | 8072 (97) |  |
| Missing | **54** | **8** | **23** | **23** |  |
| **family relationships** |  |  |  |  | $<$**0.001** |
| good | 17358 (92) | 2135 (91) | 7724 (94) | 7499 (90) |  |
| poor | 1509 (8) | 206 (9) | 457 (6) | 846 (10) |  |
| Missing | **36** | **3** | **17** | **16** |  |
| **sleeping problem** |  |  |  |  | $<$**0.001** |
| no | 17476 (93) | 1652 (71) | 7943 (97) | 7881 (95) |  |
| yes | 1336 (7) | 684 (29) | 240 (3) | 412 (5) |  |
| Missing | **91** | **8** | **15** | **68** |  |
| **sleeping disorder** |  |  |  |  | $<$**0.001** |
| no | 6380 (34) | 778 (33) | 1940 (24) | 3662 (44) |  |
| yes | 12499 (66) | 1563 (67) | 6251 (76) | 4685 (56) |  |
| Missing | **24** | **3** | **7** | **14** |  |
| **reduced sleep** |  |  |  |  | $<$**0.001** |
| no | 17477 (93) | 1653 (71) | 7943 (97) | 7881 (95) |  |
| yes | 1333 (7) | 683 (29) | 239 (3) | 411 (5) |  |
| Missing | **93** | **8** | **16** | **69** |  |
| **difficulty falling asleep** |  |  |  |  | $<$**0.001** |
| no | 8163 (43) | 1063 (45) | 2717 (33) | 4383 (53) |  |
| yes | 10691 (57) | 1277 (55) | 5474 (67) | 3940 (47) |  |
| Missing | **49** | **4** | **7** | **38** |  |
| **dreaminess** |  |  |  |  | $<$**0.001** |
| no | 16135 (86) | 2039 (87) | 6270 (77) | 7826 (94) |  |
| yes | 2711 (14) | 300 (13) | 1921 (23) | 490 (6) |  |
| Missing | **57** | **5** | **7** | **45** |  |
| **sleep disturbance** |  |  |  |  | $<$**0.001** |
| no | 14271 (76) | 1850 (79) | 5434 (66) | 6987 (84) |  |
| yes | 4580 (24) | 489 (21) | 2758 (34) | 1333 (16) |  |
| Missing | **52** | **5** | **6** | **41** |  |
| **early awakening** |  |  |  |  | $<$**0.001** |
| no | 13356 (71) | 1673 (72) | 4693 (57) | 6990 (84) |  |
| yes | 5497 (29) | 666 (28) | 3498 (43) | 1333 (16) |  |
| Missing | **50** | **5** | **7** | **38** |  |
| **other sleeping problems** |  |  |  |  | $<$**0.001** |
| no | 17849 (95) | 2137 (91) | 7775 (95) | 7937 (95) |  |
| yes | 1038 (5) | 206 (9) | 416 (5) | 416 (5) |  |
| Missing | **16** | **1** | **7** | **8** |  |
| **sleeping pills history** |  |  |  |  | 0.21 |
| no | 18862 (100) | 2338 (100) | 8176 (100) | 8348 (100) |  |
| yes | 39 (0) | 5 (0) | 22 (0) | 12 (0) |  |
| Missing | **2** | **1** | **0** | **1** |  |
| **sweat** |  |  |  |  | $<$**0.001** |
| no | 15753 (83) | 2036 (87) | 5714 (70) | 8003 (96) |  |
| yes | 3138 (17) | 307 (13) | 2480 (30) | 351 (4) |  |
| Missing | **12** | **1** | **4** | **7** |  |
| **palpitation** |  |  |  |  | $<$**0.001** |
| no | 13751 (73) | 1798 (77) | 4179 (51) | 7774 (93) |  |
| yes | 5140 (27) | 545 (23) | 4015 (49) | 580 (7) |  |
| Missing | **12** | **1** | **4** | **7** |  |
| **somatic symptoms** |  |  |  |  | $<$**0.001** |
| no | 13827 (74) | 1820 (78) | 4225 (52) | 7782 (94) |  |
| yes | 4940 (26) | 509 (22) | 3944 (48) | 487 (6) |  |
| Missing | **136** | **15** | **29** | **92** |  |
| **indecisive** |  |  |  |  | $<$**0.001** |
| no | 17044 (90) | 2103 (90) | 6748 (82) | 8193 (98) |  |
| yes | 1848 (10) | 240 (10) | 1447 (18) | 161 (2) |  |
| Missing | **11** | **1** | **3** | **7** |  |
| **individual intention** |  |  |  |  | $<$**0.001** |
| extroversion | 7085 (38) | 1100 (48) | 3595 (45) | 2390 (29) |  |
| introversion | 11432 (62) | 1201 (52) | 4463 (55) | 5768 (71) |  |
| Missing | **386** | **43** | **140** | **203** |  |
| **personality disorders** |  |  |  |  | 0.42 |
| no | 18889 (100) | 2341 (100) | 8191 (100) | 8357 (100) |  |
| yes | 13 (0) | 3 (0) | 6 (0) | 4 (0) |  |
| Missing | **1** | **0** | **1** | **0** |  |
| **special personality** |  |  |  |  | $<$**0.001** |
| no | 18643 (99) | 2288 (98) | 8067 (98) | 8288 (99) |  |
| yes | 259 (1) | 56 (2) | 131 (2) | 72 (1) |  |
| Missing | **1** | **0** | **0** | **1** |  |
| **impulsive behaviors** |  |  |  |  | $<$**0.001** |
| no | 16410 (87) | 1905 (81) | 7669 (94) | 6836 (82) |  |
| yes | 2492 (13) | 439 (19) | 529 (6) | 1524 (18) |  |
| Missing | **1** | **0** | **0** | **1** |  |
| **self harm** |  |  |  |  | $<$**0.001** |
| no | 14679 (78) | 1708 (73) | 5802 (71) | 7169 (86) |  |
| yes | 4223 (22) | 636 (27) | 2396 (29) | 1191 (14) |  |
| Missing | **1** | **0** | **0** | **1** |  |
| **suicide** |  |  |  |  | $<$**0.001** |
| no ideas or behaviors | 13292 (70) | 1582 (67) | 4274 (52) | 7436 (89) |  |
| yes | 5610 (30) | 762 (33) | 3923 (48) | 925 (11) |  |
| Missing | **1** | **0** | **1** | **0** |  |

**Table S4 RDoC domain characteristics of various DSM-5 categories**

| **Covariate** | **Full Sample (n=26081)** | **BD (n=3258)** | **MDD (n=13075)** | **SCZ (n=9748)** | **p-value** |
| --- | --- | --- | --- | --- | --- |
| **negative domain** |  |  |  |  | $<$**0.001** |
| Mean (sd) | 3.4 (2.5) | 3.8 (2.6) | 4.1 (2.6) | 2.4 (1.8) |  |
| Median (Min,Max) | 3.4 (0,16.9) | 3.4 (0,15.7) | 3.4 (0,16.9) | 2.2 (0,13.5) |  |
| **positive domain** |  |  |  |  | $<$**0.001** |
| Mean (sd) | 4.2 (2.8) | 4.8 (2.9) | 4.6 (2.9) | 3.6 (2.4) |  |
| Median (Min,Max) | 3.3 (0,19.7) | 4.9 (0,18) | 4.9 (0,19.7) | 3.3 (0,18) |  |
| **cognitive domain** |  |  |  |  | $<$**0.001** |
| Mean (sd) | 5.2 (3) | 5.3 (3) | 4.8 (3) | 5.8 (2.8) |  |
| Median (Min,Max) | 4.9 (0,18.3) | 4.9 (0,17.1) | 4.9 (0,18.3) | 6.1 (0,17.1) |  |
| **social domain** |  |  |  |  | $<$**0.001** |
| Mean (sd) | 3.7 (2.8) | 3.7 (2.7) | 3.2 (2.6) | 4.6 (2.8) |  |
| Median (Min,Max) | 3.8 (0,18.9) | 3.8 (0,17) | 1.9 (0,17) | 3.8 (0,18.9) |  |
| **arousal domain** |  |  |  |  | $<$**0.001** |
| Mean (sd) | 4 (2.2) | 4.4 (2.3) | 4.2 (2.3) | 3.6 (1.9) |  |
| Median (Min,Max) | 3.8 (0,16.2) | 3.8 (0,16.2) | 3.8 (0,15.2) | 3.8 (0,14.3) |  |

**Table S5 The characteristics of Peripheral blood test in different DSM-5 categories**

| **Covariate** | **Full Sample (n=26081)** | **BD (n=3258)** | **MDD (n=13075)** | **SCZ (n=9748)** | **p-value** |
| --- | --- | --- | --- | --- | --- |
| **Basophil percentage** |  |  |  |  | $<$**0.001** |
| z | 24273 (95) | 3036 (95) | 12007 (95) | 9230 (96) |  |
| h | 1171 (5) | 150 (5) | 676 (5) | 345 (4) |  |
| Mean |  |  |  |  |  |
| Missing | **637** | **72** | **392** | **173** |  |
| **CRP** |  |  |  |  | $<$**0.001** |
| z | 1191 (67) | 131 (66) | 825 (72) | 235 (54) |  |
| h | 586 (33) | 67 (34) | 318 (28) | 201 (46) |  |
| Mean |  |  |  |  |  |
| Missing | **24304** | **3060** | **11932** | **9312** |  |
| **Eosinophil percentage** |  |  |  |  | $<$**0.001** |
| z | 22852 (90) | 2907 (91) | 11810 (93) | 8135 (85) |  |
| h | 704 (3) | 100 (3) | 335 (3) | 269 (3) |  |
| l | 1919 (8) | 184 (6) | 556 (4) | 1179 (12) |  |
| Mean |  |  |  |  |  |
| Missing | **606** | **67** | **374** | **165** |  |
| **Lymphocyte count** |  |  |  |  | $<$**0.001** |
| z | 22748 (89) | 2920 (91) | 11304 (89) | 8524 (89) |  |
| h | 768 (3) | 125 (4) | 344 (3) | 299 (3) |  |
| l | 1975 (8) | 149 (5) | 1059 (8) | 767 (8) |  |
| Mean |  |  |  |  |  |
| Missing | **590** | **64** | **368** | **158** |  |
| **Lymphocyte percentage** |  |  |  |  | $<$**0.001** |
| z | 21167 (83) | 2687 (84) | 10909 (86) | 7571 (79) |  |
| h | 1182 (5) | 182 (6) | 638 (5) | 362 (4) |  |
| l | 3144 (12) | 325 (10) | 1162 (9) | 1657 (17) |  |
| Mean |  |  |  |  |  |
| Missing | **588** | **64** | **366** | **158** |  |
| **Monocyte count** |  |  |  |  | $<$**0.001** |
| z | 23134 (91) | 2804 (88) | 11798 (93) | 8532 (89) |  |
| h | 2288 (9) | 387 (12) | 861 (7) | 1040 (11) |  |
| l | 64 (0) | 3 (0) | 44 (0) | 17 (0) |  |
| Mean |  |  |  |  |  |
| Missing | **595** | **64** | **372** | **159** |  |
| **Monocyte percentage** |  |  |  |  | **0.003** |
| z | 23712 (93) | 2932 (92) | 11834 (93) | 8946 (93) |  |
| h | 1531 (6) | 238 (7) | 750 (6) | 543 (6) |  |
| l | 244 (1) | 24 (1) | 120 (1) | 100 (1) |  |
| Mean |  |  |  |  |  |
| Missing | **594** | **64** | **371** | **159** |  |
| **Neutrophil count norm** |  |  |  |  | $<$**0.001** |
| h | 729 (3) | 78 (2) | 195 (2) | 456 (5) |  |
| l | 742 (3) | 103 (3) | 482 (4) | 157 (2) |  |
| z | 23961 (94) | 3003 (94) | 12000 (95) | 8958 (94) |  |
| Mean |  |  |  |  |  |
| Missing | **649** | **74** | **398** | **177** |  |
| **Neutrophil percentage norm** |  |  |  |  | $<$**0.001** |
| h | 2000 (8) | 189 (6) | 671 (5) | 1140 (12) |  |
| l | 5584 (22) | 859 (27) | 3208 (25) | 1517 (16) |  |
| z | 17848 (70) | 2136 (67) | 8798 (69) | 6914 (72) |  |
| Mean |  |  |  |  |  |
| Missing | **649** | **74** | **398** | **177** |  |
| **Platelet count** |  |  |  |  | $<$**0.001** |
| z | 22430 (88) | 2799 (88) | 11207 (88) | 8424 (88) |  |
| h | 2111 (8) | 306 (10) | 949 (7) | 856 (9) |  |
| l | 952 (4) | 89 (3) | 553 (4) | 310 (3) |  |
| Mean |  |  |  |  |  |
| Missing | **588** | **64** | **366** | **158** |  |
| **Platelet distribution width** |  |  |  |  | 0.46 |
| z | 9319 (83) | 1052 (83) | 3919 (83) | 4348 (84) |  |
| h | 544 (5) | 52 (4) | 239 (5) | 253 (5) |  |
| l | 1341 (12) | 163 (13) | 575 (12) | 603 (12) |  |
| Mean |  |  |  |  |  |
| Missing | **14877** | **1991** | **8342** | **4544** |  |
| **White blood cell leukocyte count** |  |  |  |  | $<$**0.001** |
| z | 22960 (90) | 2900 (91) | 11597 (91) | 8463 (88) |  |
| h | 1565 (6) | 213 (7) | 445 (4) | 907 (9) |  |
| l | 968 (4) | 81 (3) | 667 (5) | 220 (2) |  |
| Mean |  |  |  |  |  |
| Missing | **588** | **64** | **366** | **158** |  |
| **NLR norm** |  |  |  |  | $<$**0.001** |
| z | 22135 (87) | 2809 (88) | 11292 (89) | 8034 (84) |  |
| h | 2393 (9) | 229 (7) | 838 (7) | 1326 (14) |  |
| l | 904 (4) | 146 (5) | 547 (4) | 211 (2) |  |
| Mean |  |  |  |  |  |
| Missing | **649** | **74** | **398** | **177** |  |
| **MLR norm** |  |  |  |  | $<$**0.001** |
| h | 152 (1) | 12 (0) | 61 (0) | 79 (1) |  |
| l | 11737 (46) | 1491 (47) | 6193 (49) | 4053 (42) |  |
| z | 13597 (53) | 1691 (53) | 6449 (51) | 5457 (57) |  |
| Mean |  |  |  |  |  |
| Missing | **595** | **64** | **372** | **159** |  |
| **PLR norm** |  |  |  |  | $<$**0.001** |
| h | 330 (1) | 33 (1) | 148 (1) | 149 (2) |  |
| l | 10216 (40) | 1388 (43) | 5032 (40) | 3796 (40) |  |
| z | 14945 (59) | 1773 (56) | 7527 (59) | 5645 (59) |  |
| mean |  |  |  |  |  |
| Missing | **590** | **64** | **368** | **158** |  |
| **Hematocrit** |  |  |  |  | $<$**0.001** |
| z | 22000 (86) | 2814 (88) | 10940 (86) | 8246 (86) |  |
| h | 983 (4) | 111 (3) | 432 (3) | 440 (5) |  |
| l | 2506 (10) | 269 (8) | 1334 (10) | 903 (9) |  |
| Mean |  |  |  |  |  |
| Missing | **592** | **64** | **369** | **159** |  |
| **Red blood cell count** |  |  |  |  | $<$**0.001** |
| z | 22191 (87) | 2851 (89) | 10872 (86) | 8468 (88) |  |
| h | 1054 (4) | 122 (4) | 466 (4) | 466 (5) |  |
| l | 2248 (9) | 221 (7) | 1371 (11) | 656 (7) |  |
| Mean |  |  |  |  |  |
| Missing | **588** | **64** | **366** | **158** |  |
| **Mean corpuscular hemoglobin concentration** |  |  |  |  | $<$**0.001** |
| z | 22352 (88) | 2776 (87) | 11085 (87) | 8491 (89) |  |
| h | 294 (1) | 25 (1) | 144 (1) | 125 (1) |  |
| l | 2844 (11) | 393 (12) | 1478 (12) | 973 (10) |  |
| Missing | **591** | **64** | **368** | **159** |  |
| **Mean corpuscular hemoglobin** |  |  |  |  | $<$**0.001** |
| z | 23049 (90) | 2890 (90) | 11415 (90) | 8744 (91) |  |
| h | 537 (2) | 49 (2) | 300 (2) | 188 (2) |  |
| l | 1907 (7) | 255 (8) | 994 (8) | 658 (7) |  |
| Missing | **588** | **64** | **366** | **158** |  |
| **Mean corpuscular volume** |  |  |  |  | $<$**0.001** |
| z | 23345 (92) | 2934 (92) | 11509 (91) | 8902 (93) |  |
| al | 1 (0) | 0 (0) | 1 (0) | 0 (0) |  |
| h | 720 (3) | 78 (2) | 446 (4) | 196 (2) |  |
| l | 1427 (6) | 182 (6) | 753 (6) | 492 (5) |  |
| Missing | **588** | **64** | **366** | **158** |  |
| **Direct LDL** |  |  |  |  | $<$**0.001** |
| z | 24412 (98) | 3064 (98) | 12014 (97) | 9334 (98) |  |
| h | 569 (2) | 54 (2) | 358 (3) | 157 (2) |  |
| Missing | **1100** | **140** | **703** | **257** |  |
| **Triglycerides** |  |  |  |  | **0.012** |
| z | 21934 (86) | 2756 (86) | 10844 (85) | 8334 (87) |  |
| h | 3512 (14) | 440 (14) | 1838 (14) | 1234 (13) |  |
| l | 32 (0) | 3 (0) | 14 (0) | 15 (0) |  |
| Missing | **603** | **59** | **379** | **165** |  |
| **Total cholesterol** |  |  |  |  | $<$**0.001** |
| z | 23030 (90) | 2922 (91) | 11429 (90) | 8679 (91) |  |
| h | 1344 (5) | 130 (4) | 837 (7) | 377 (4) |  |
| l | 1104 (4) | 147 (5) | 430 (3) | 527 (5) |  |
| Missing | **603** | **59** | **379** | **165** |  |
| **Albumin** |  |  |  |  | $<$**0.001** |
| z | 21495 (84) | 2732 (85) | 10415 (82) | 8348 (87) |  |
| h | 83 (0) | 9 (0) | 14 (0) | 60 (1) |  |
| l | 3925 (15) | 459 (14) | 2285 (18) | 1181 (12) |  |
| Missing | **578** | **58** | **361** | **159** |  |
| **Alanine aminotransferase** |  |  |  |  | $<$**0.001** |
| z | 23282 (91) | 2942 (92) | 11751 (92) | 8589 (90) |  |
| al | 51 (0) | 9 (0) | 32 (0) | 10 (0) |  |
| h | 2170 (9) | 249 (8) | 931 (7) | 990 (10) |  |
| Missing | **578** | **58** | **361** | **159** |  |
| **Glutamyltransferase** |  |  |  |  | 0.17 |
| z | 23863 (94) | 3012 (94) | 11863 (93) | 8988 (94) |  |
| h | 1611 (6) | 185 (6) | 840 (7) | 586 (6) |  |
| l | 29 (0) | 3 (0) | 11 (0) | 15 (0) |  |
| Missing | **578** | **58** | **361** | **159** |  |
| **Aspartate aminotransferase** |  |  |  |  | $<$**0.001** |
| z | 23836 (93) | 2980 (93) | 12013 (94) | 8843 (92) |  |
| al | 1 (0) | 1 (0) | 0 (0) | 0 (0) |  |
| h | 1666 (7) | 219 (7) | 701 (6) | 746 (8) |  |
| Missing | **578** | **58** | **361** | **159** |  |
| **Direct bilirubin** |  |  |  |  | $<$**0.001** |
| z | 24710 (97) | 3131 (98) | 12446 (98) | 9133 (95) |  |
| al | 28 (0) | 2 (0) | 16 (0) | 10 (0) |  |
| h | 765 (3) | 67 (2) | 252 (2) | 446 (5) |  |
| Missing | **578** | **58** | **361** | **159** |  |
| **Total bilirubin** |  |  |  |  | $<$**0.001** |
| z | 22959 (90) | 2829 (88) | 11529 (91) | 8601 (90) |  |
| al | 0 (0) | 0 (0) | 0 (0) | 0 (0) |  |
| h | 489 (2) | 38 (1) | 195 (2) | 256 (3) |  |
| l | 2055 (8) | 333 (10) | 990 (8) | 732 (8) |  |
| Missing | **578** | **58** | **361** | **159** |  |
| **Creatinine** |  |  |  |  | $<$**0.001** |
| z | 24586 (96) | 3068 (96) | 12241 (96) | 9277 (97) |  |
| h | 333 (1) | 64 (2) | 199 (2) | 70 (1) |  |
| l | 584 (2) | 68 (2) | 274 (2) | 242 (3) |  |
| Missing | **578** | **58** | **361** | **159** |  |
| **Cystatin C** |  |  |  |  | $<$**0.001** |
| z | 24230 (95) | 3096 (97) | 11973 (94) | 9161 (96) |  |
| h | 1193 (5) | 98 (3) | 704 (6) | 391 (4) |  |
| l | 34 (0) | 5 (0) | 13 (0) | 16 (0) |  |
| Missing | **624** | **59** | **385** | **180** |  |
| **Total protein** |  |  |  |  | $<$**0.001** |
| z | 19303 (76) | 2425 (76) | 9195 (72) | 7683 (80) |  |
| h | 102 (0) | 6 (0) | 29 (0) | 67 (1) |  |
| l | 6098 (24) | 769 (24) | 3490 (27) | 1839 (19) |  |
| Missing | **578** | **58** | **361** | **159** |  |
| **Urate** |  |  |  |  | $<$**0.001** |
| z | 22354 (88) | 2763 (86) | 11379 (89) | 8212 (86) |  |
| h | 2232 (9) | 354 (11) | 792 (6) | 1086 (11) |  |
| l | 917 (4) | 83 (3) | 543 (4) | 291 (3) |  |
| Missing | **578** | **58** | **361** | **159** |  |
| **Urea** |  |  |  |  | $<$**0.001** |
| z | 22702 (89) | 2846 (89) | 11464 (90) | 8392 (88) |  |
| h | 678 (3) | 79 (2) | 279 (2) | 320 (3) |  |
| l | 2123 (8) | 275 (9) | 971 (8) | 877 (9) |  |
| Missing | **578** | **58** | **361** | **159** |  |
| **Calcium** |  |  |  |  | **0.02** |
| z | 23859 (94) | 3015 (94) | 11828 (93) | 9016 (94) |  |
| h | 71 (0) | 10 (0) | 36 (0) | 25 (0) |  |
| l | 1524 (6) | 171 (5) | 823 (6) | 530 (6) |  |
| Missing | **627** | **62** | **388** | **177** |  |
| **Alkaline phosphatase** |  |  |  |  | $<$**0.001** |
| z | 21590 (85) | 2684 (84) | 10530 (83) | 8376 (87) |  |
| h | 667 (3) | 73 (2) | 245 (2) | 349 (4) |  |
| l | 3246 (13) | 443 (14) | 1939 (15) | 864 (9) |  |
| Missing | **578** | **58** | **361** | **159** |  |
| **Glucose** |  |  |  |  | $<$**0.001** |
| z | 22001 (86) | 2732 (85) | 11387 (90) | 7882 (82) |  |
| h | 2663 (10) | 334 (10) | 886 (7) | 1443 (15) |  |
| l | 845 (3) | 133 (4) | 447 (4) | 265 (3) |  |
| Missing | **572** | **59** | **355** | **158** |  |

**
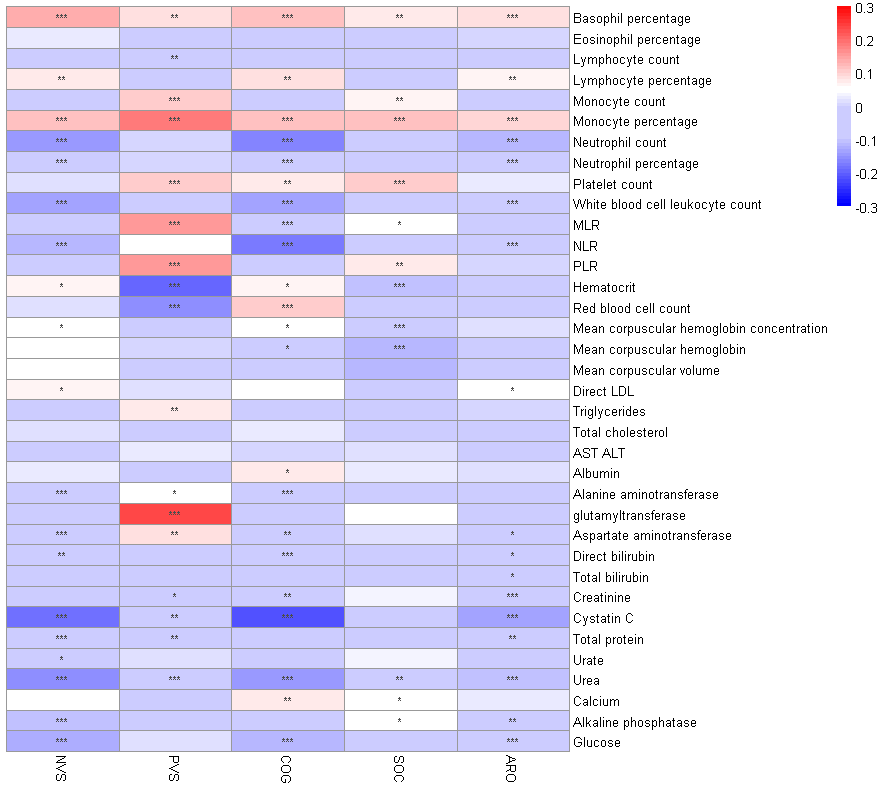
**

**Fig. S1 Heatmap showing adjusted effect sizes (*ß*) for associations between peripheral blood test index with RDoC domain scores in MDD population**

**
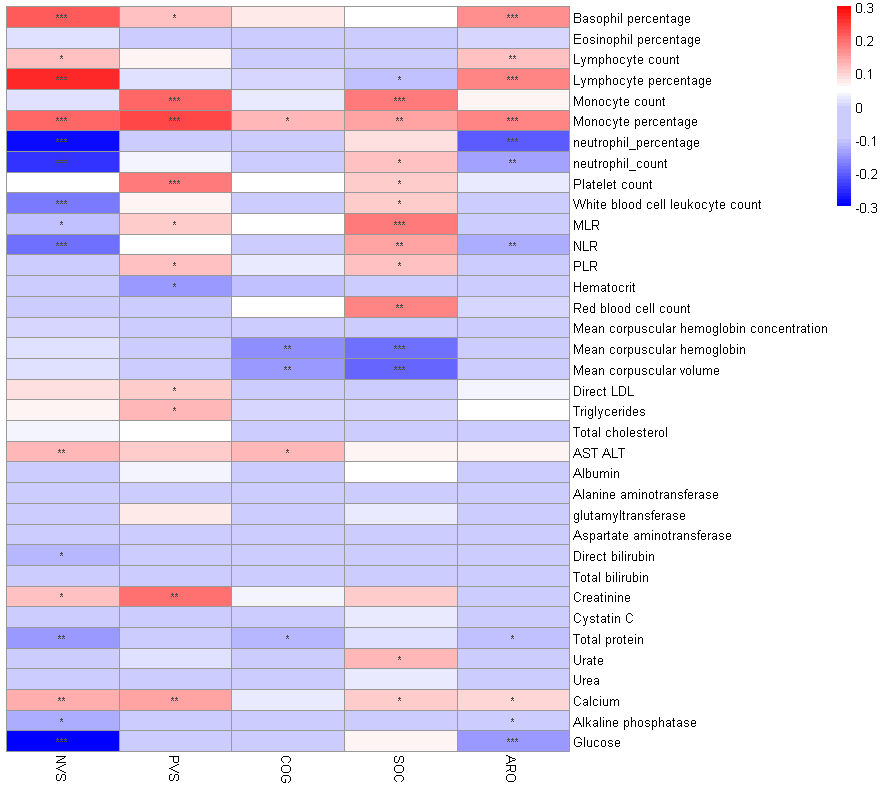
**

**Fig.S2 Heatmap showing adjusted effect sizes (*ß*) for associations between peripheral blood test index with RDoC domain scores in BD population**

**
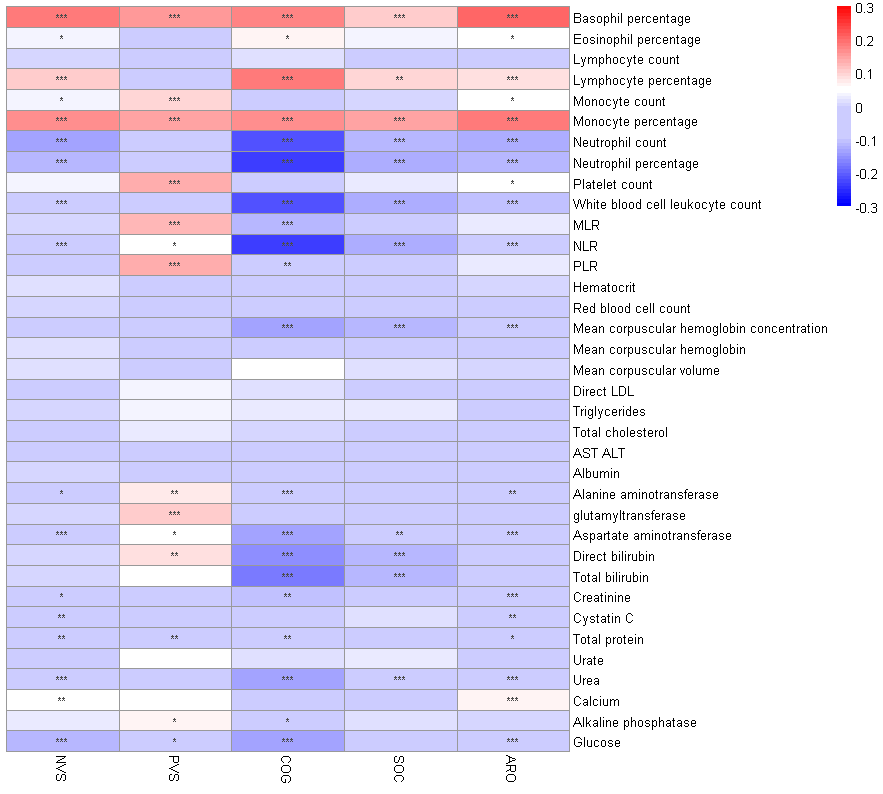
**

**Fig.S3 Heatmap showing adjusted effect sizes (*ß*) for associations between peripheral blood test index with RDoC domain scores in SCZ population**

**
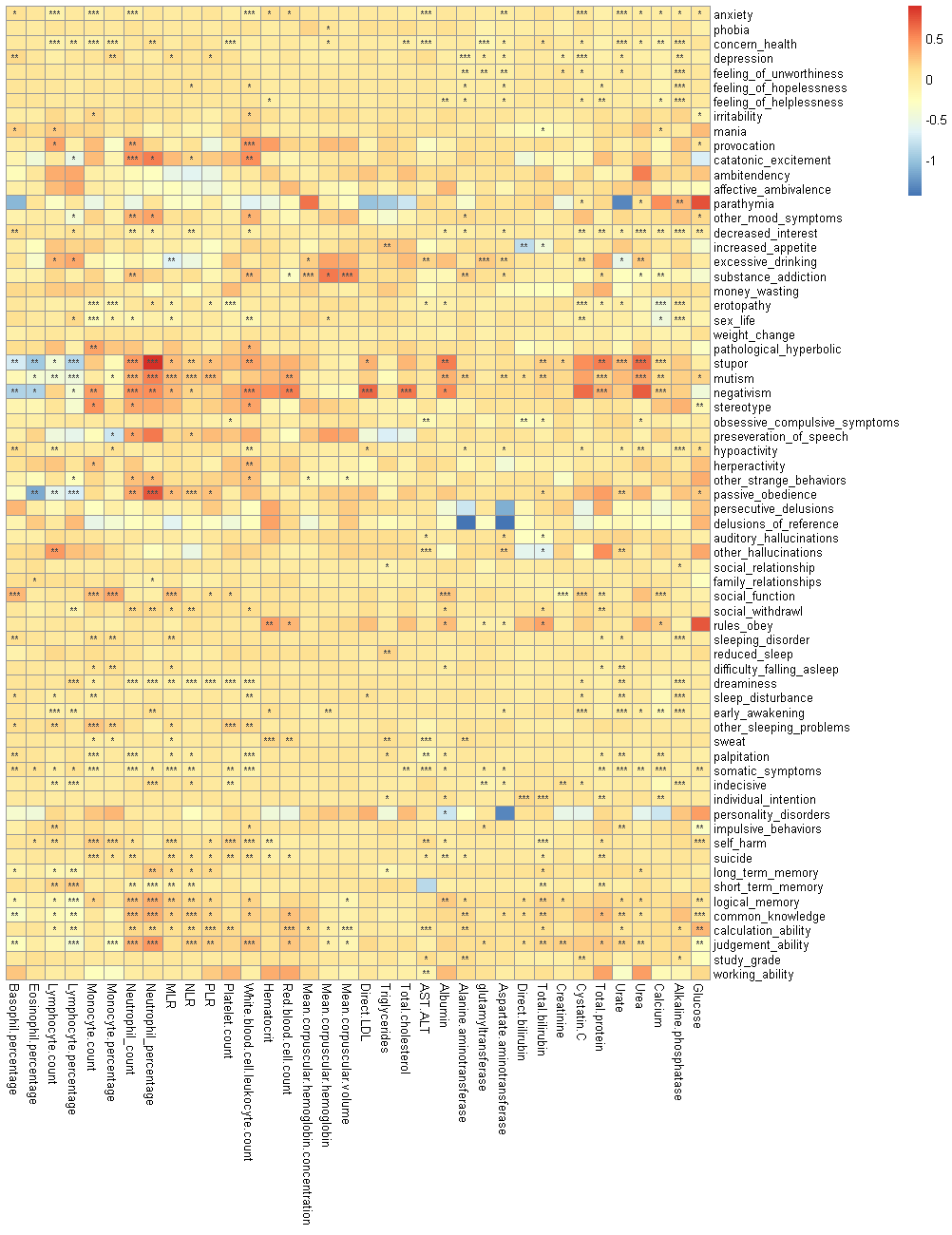
**

**Fig.S4 The association between peripheral blood test index with different symptoms in MDD population**

**
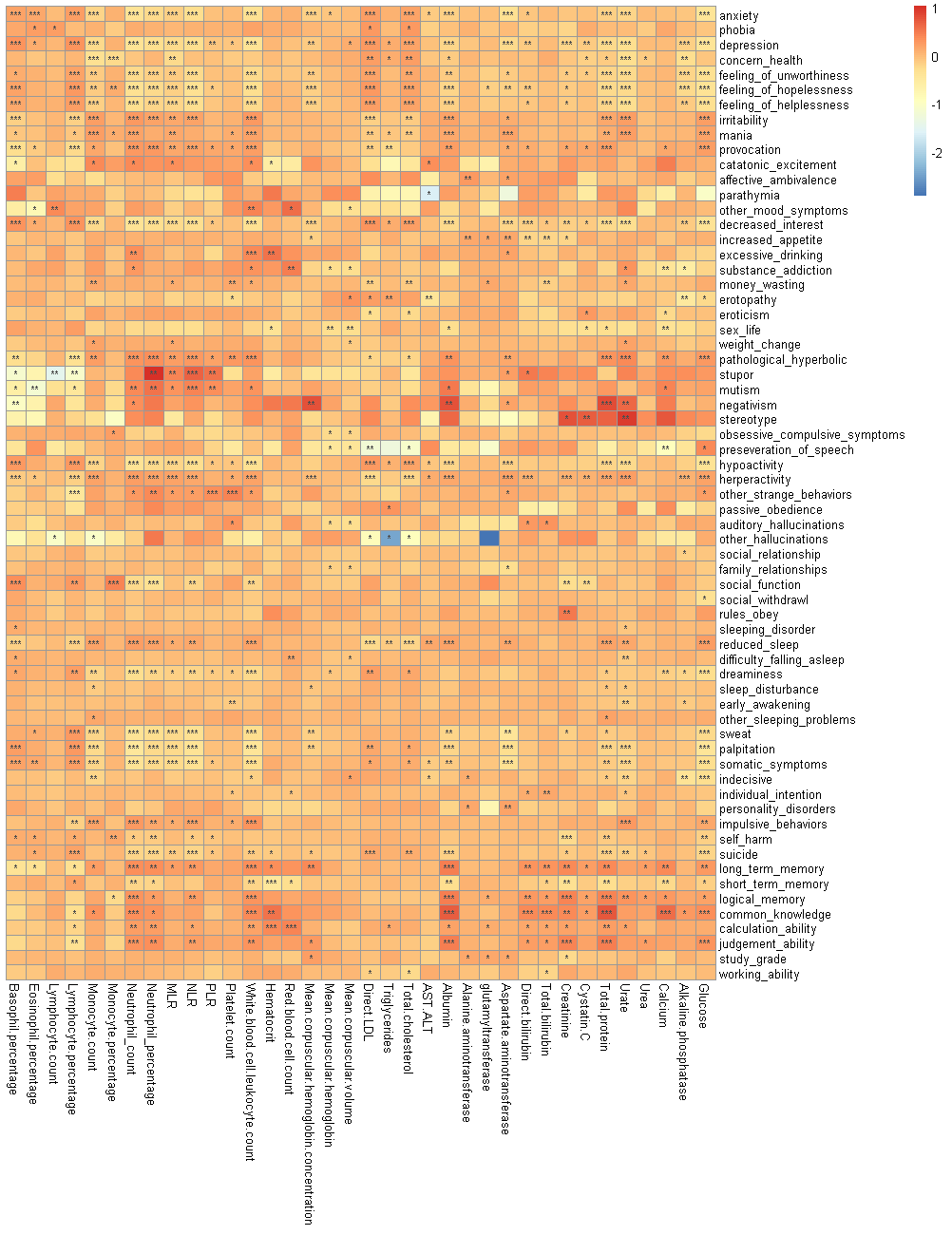
**

**Fig.S5 The association between peripheral blood test index with different symptoms in BD population**

**
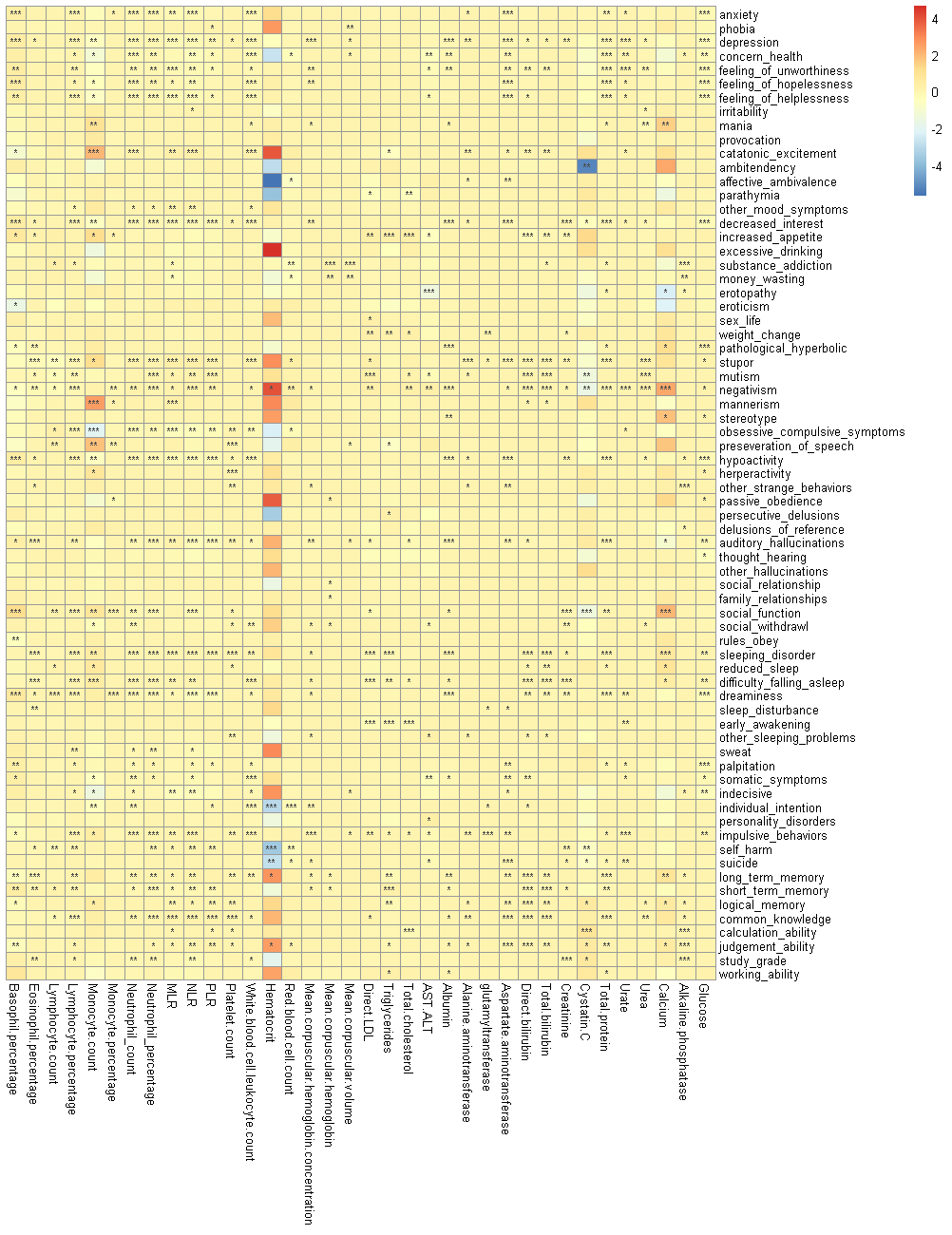
**

**Fig.S6 The association between peripheral blood test index with different symptoms in SCZ population**
